# Enteric Infection-Associated Reactive Arthritis: A Systematic Review and Meta-Analysis

**DOI:** 10.3390/jcm13123433

**Published:** 2024-06-12

**Authors:** Darya Shafiee, Zhandos Salpynov, Arnur Gusmanov, Yerkhanat Khuanbai, Zhussipbek Mukhatayev, Jeannette Kunz

**Affiliations:** 1 Department of Biomedical Sciences, School of Medicine, Nazarbayev University, Astana 020000, Kazakhstan; darya.chunikhina@nu.edu.kz (D.S.); zhandos.salpynov@nu.edu.kz (Z.S.); arnur.gusmanov@nu.edu.kz (A.G.); zhussipbek.mukhatayev@nu.edu.kz (Z.M.); 2 National Laboratory Astana, Astana 020000, Kazakhstan; dr.yerkhanat@gmail.com

**Keywords:** reactive arthritis, postinfectious arthritis, *Campylobacter*, *Escherichia*, *Salmonella*, *Shigella*, *Yersinia*, systematic review, meta-analysis

## Abstract

**Background.** The objective of this systematic review and meta-analysis was to estimate the proportions of individuals infected with *Campylobacter*, *Escherichia*, *Salmonella*, *Shigella*, or *Yersinia* who develop reactive arthritis. **Methods.** A systematic review was conducted, encompassing English-language articles published before January 2024, sourced from the Embase, PubMed, Scopus, and Web of Science databases. This review included observational studies that reported the occurrence of reactive arthritis (ReA) among patients with *Campylobacter*, *Escherichia*, *Salmonella*, *Shigella*, or *Yersinia* infections. Data extraction was carried out independently by two reviewers. Subsequently, a random-effects meta-analysis was performed, with heterogeneity assessed using the I^2^ value. Additionally, meta-regression was employed to investigate the potential influence of study-level variables on the observed heterogeneity. **Results.** A total of 87 studies were identified; 23 reported on ReA development after *Campylobacter* infection, 7 reported on ReA after *Escherichia infection*, 30 reported ReA onset after salmonellosis, 14 reported ReA after shigellosis, and 13 reported ReA after *Yersinia* infection. The proportion of *Campylobacter* patients who developed ReA was 0.03 (95% CI [0.01, 0.06], I^2^ = 97.62%); the proportion of *Escherichia* patients who developed ReA was 0.01 (95% CI [0.00, 0.06], I^2^ = 92.78%); the proportion of *Salmonella* patients was 0.04 (95% CI [0.02, 0.08], I^2^ = 97.67%); the proportion of *Shigella* patients was 0.01 (95% CI [0.01, 0.03], I^2^ = 90.64%); and the proportion of *Yersinia* patients who developed ReA was 0.05 (95% CI [0.02, 0.13], I^2^ = 96%). **Conclusion.** A significant proportion of *Salmonella*, *Shigella*, and *Yersinia* cases resulted in ReA. Nonetheless, it is important to interpret the findings cautiously due to the substantial heterogeneity observed between studies.

## 1. Introduction

Reactive arthritis (ReA) is an inflammatory form of arthritis classified as seronegative spondyloarthropathies with frequent genetic HLA-B27 predisposition [1]. Disease manifestations are quite diverse, as ReA affects articular (joints and entheses) and extraarticular (ocular, mucosal, cutaneous, and cardiac) sites [2]. The joint symptoms can vary from mild mono- or oligoarthralgia to highly disabling polyarthritis [3]. ReA typically impacts the joints of the lower extremities, particularly the knees and ankles, although it may not necessarily involve the axial region. It can also affect small joints, with or without redness and swelling, or can present as tenosynovitis [4]. The duration of symptoms is usually 6 months, and symptoms may occur intermittently, leading to long-term illness in some individuals (10–30% of patients) [5]. Diagnosis is complicated because imaging results are inconclusive, and no specific laboratory test exists to date [3]. ReA can be easily misdiagnosed and underreported due to its various manifestations, and in this case, the absence of specific treatment may result in serious complications such as chronic destructive arthritis and other disabling disease sequelae [4].

Acute ReA is marked by sterile joints and usually appears within four weeks after an intestinal infection. The development of ReA has been associated with several gastrointestinal infections, such as infections caused by *Campylobacter jejuni*, *Escherichia coli O157:H7*, *Salmonella enteritidis*, *Shigella flexneri*, *Shigella dysenteriae*, and *Yersinia enterocilitica*, which are the most common enteric pathogens known to cause ReA [6]. ReA can also result from urogenital infections triggered by pathogens such as *Chlamydia trachomatis*, *Gardnerella vaginalis*, *Mycoplasma genitalium*, *Neisseria gonorrheae*, and *Ureaplasma urealyticum* [7]. Moreover, other probable bacterial triggers of ReA development have been reported, including *Bacillus cereus*, *Bartonella species*, *Borrelia burgdorferi* (the causative agent of Lyme disease), *Brucella abortus* (causing brucellosis), *Clostridium difficile* (associated with antibiotic-associated diarrhea), *Helicobacter pylori* (linked to gastric ulcers), and *Pseudomonas* species [8]. Several cases of ReA development were reported after SARS-CoV-2 infection in pediatric patients [9,10] as well as in older adults [11,12,13,14]. Several cases of reactive arthritis have also been described after the administration of the Bacillus Calmette–Guerin (BCG) vaccine [15].

In 2022, the European Centre for Disease Prevention and Control reported that the prevalence of Campylobacteriosis in the general population was 46.9 cases per 100,000 people [16]. The prevalence of *Escherichia* infections was 2.5 cases per 100,000 people [17], while salmonellosis had a higher prevalence of 15.5 cases per 100,000 people [18]. Shigellosis has a prevalence of 1.5 per 100,000 individuals [19], and yersiniosis has a prevalence of 2.2 per 100,000 individuals [20].

In the general population, reactive arthritis is quite rare, and the incidence of this disease reaches 27 cases per 100,000 people, as concluded from population-based studies [21]. The female–male ratio of ReA is 1:3, and the disease severity in women is considerably lower than that in men [22,23]. White patients are more prone to developing severe ReA, likely due to the increased prevalence of the HLA-B27 allele [24].

The aim of this study was to conduct a comprehensive systematic review and meta-analysis focused on estimating the proportion of ReA following enteric infections caused by five specific foodborne bacterial pathogens: *Campylobacter* spp., *Escherichia coli O157:H7*, *nontyphoidal Salmonella*, *Shigella* spp., and *Yersinia* spp. These pathogens were selected based on substantial evidence linking them to ReA development, as documented in the literature [25,26]. The findings of this research are expected to contribute valuable insights that can improve evidence-based patient management by raising awareness among clinicians about ReA following infections with these specific bacterial pathogens. This targeted approach will help inform clinical decision-making and facilitate early recognition and appropriate management of ReA cases associated with enteric infections caused by these identified pathogens.

## 2. Materials and Methods

### 2.1. Search Strategy

We performed a literature search of published articles in four publicly available databases: Embase, PubMed, Scopus, and Web of Science. The search focused on the keywords “reactive arthritis” and “*Campylobacter*”, “*Escherichia*”, “*Salmonella*”, “*Shigella*”, or “*Yersinia*” (the specific keyword combinations used for database searches are detailed in Table S1). We conducted searches for articles published up to and including January 2024. This broad timeframe was chosen due to the limited availability of research papers on the reviewed topic, necessitating a comprehensive search across multiple years.

Articles with confirmed *Campylobacter*, *Escherichia*, *Salmonella*, *Shigella*, or *Yersinia* bacterial infection by blood or stool culture and further development of reactive arthritis within one year after infection were used. Prospective and retrospective studies confirmed ReA development via mailed questionnaires and personal or phone interviews.

**Figure 1:**
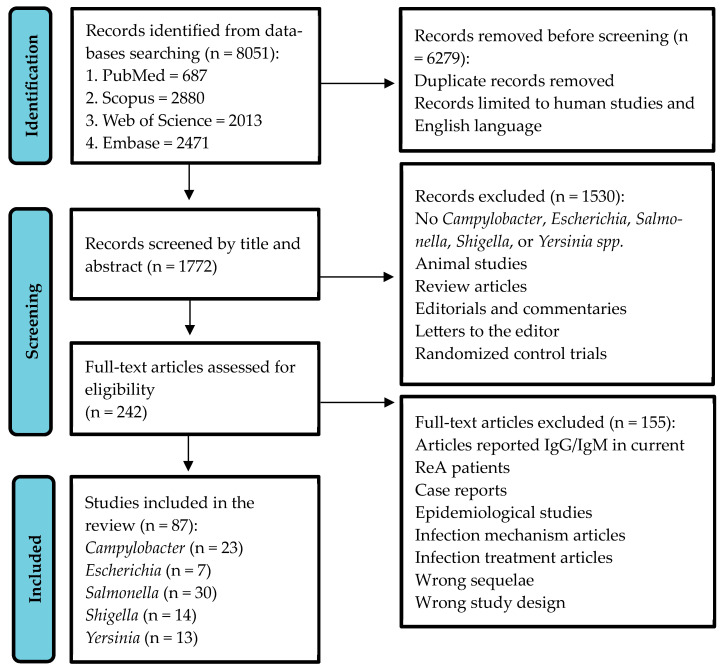
PRISMA flow chart of the process of selecting ReA studies for *Campylobacter*, *Escherichia*, *Salmonella*, *Shigella*, and *Yersinia*.

Case-control studies that focused on the search for at least one of the abovementioned bacteria in the case and control populations were also included. The initial literature search yielded a total of 8051 studies. The PRISMA flowchart of the selection process of ReA studies for *Campylobacter*, *Escherichia*, *Salmonella*, *Shigella*, and *Yersinia* is shown in Figure 1. The PRISMA checklist is detailed in Table S2.

### 2.2. Eligibility Criteria and Data Extraction

We removed duplicate records and restricted our search to articles published exclusively in English and those focused on human studies. This refinement reduced the list by 6279 publications. Two independent reviewers screened the titles and abstracts of the resulting 1772 studies. We excluded studies with inappropriate study designs, such as animal studies, case reports, review articles, editorials, commentaries, letters to the editor, conference abstracts, and randomized control trials. Studies concentrating on bacteria other than *Campylobacter*, *Escherichia*, *Salmonella*, *Shigella*, or *Yersinia* were also excluded from the search. Studies with zero cases of ReA after bacterial infection were included in the final analysis. When two researchers disagreed regarding the inclusion of studies, the corresponding author intervened to resolve the disagreement.

Following a detailed review of full-text articles, two independent reviewers further excluded studies that did not meet the predefined inclusion criteria, ensuring that the selected studies were most pertinent to our analysis (Tables S3–S7). After screening the abstracts, a total of 242 full-text articles were assessed for eligibility. Of these, 155 publications were excluded due to reports of the wrong sequelae, descriptions of the infection mechanisms and treatments, or articles that selected subjects based on ReA status and determined previous exposure to bacteria. Eventually, 87 studies met the eligibility criteria and were included in the final systematic review and meta-analysis.

### 2.3. Meta-Analysis and Risk of Bias Assessment

Logistic regression models (particularly “logit-transformed proportion”) were used to estimate the weighted proportion of ReA for single pathogens [27]. To account for both within-study and between-study variability, we employed a random-effects model with the restricted maximum-likelihood estimator in our pooled analysis [28].

In cases where a study reported zero ReA cases, we added 0.5 to the denominators and numerators for calculating pooled rates. The heterogeneity within each aggregated dataset was assessed using Cochran’s Q and Higgins I^2^ tests. High heterogeneity was deemed present if I^2^ exceeded 75% or if the *p* value of the Q test was less than 0.05. I^2^ values ranging from 0 to 50% are categorized as low, while those falling between 50% and 75% are considered moderately heterogeneous [29]. For the primary analysis involving more than 10 studies, funnel plots were used to assess publication bias. However, publication bias was not evaluated for the exploratory analysis due to the limited number of studies in each stratum [30]. All the statistical analyses were performed in STATA (version 18.0).

## 3. Results

### 3.1. Campylobacter

**Figure 2:**
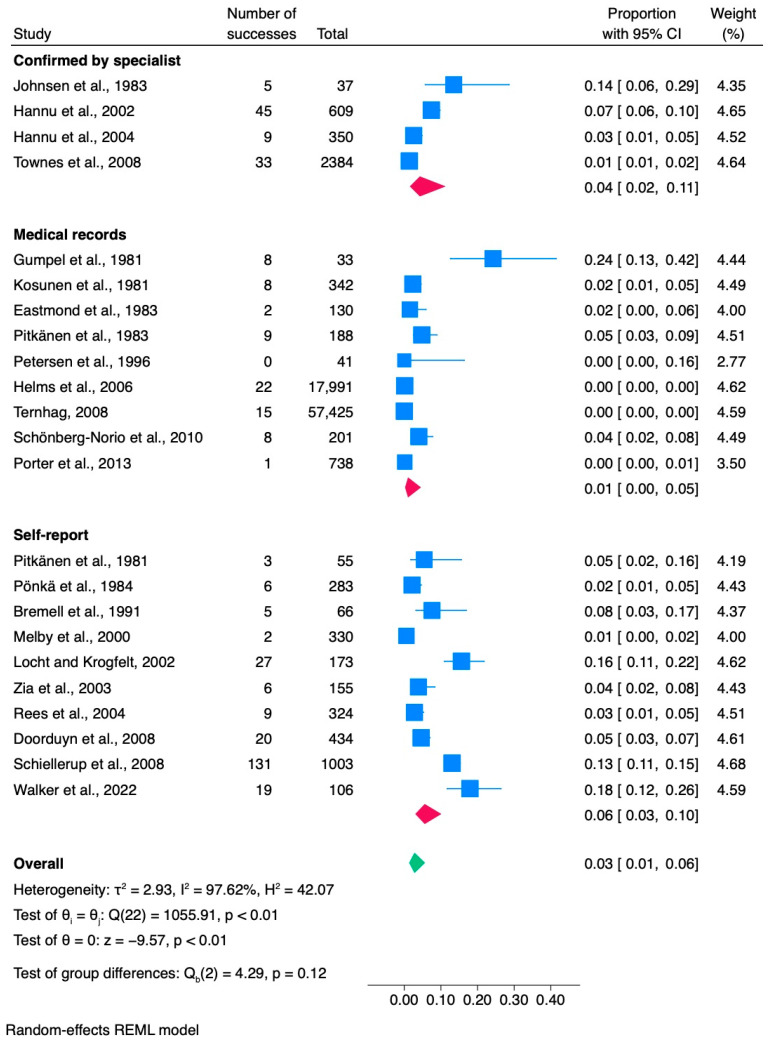
Subgroup meta-analysis of ReA cases among patients with *Campylobacter* infection [6,25,31,32,33,34,35,36,37,38,39,40,41,42,43,44,45,46,47,48,49,50,51]. CI, confidence interval.

A total of 23 studies were analyzed to determine the proportion of ReA linked to *Campylobacter* infection (Table 1). These studies included 83,398 confirmed or probable cases of campylobacteriosis, of which 393 individuals developed ReA. According to the analysis, the proportion of ReA linked to *Campylobacter* infection was 0.03, with a confidence interval of [0.01, 0.06], as indicated in Figure 2. Four studies included participants who were under the age of 18. Among the studies included in the analysis, 20 were conducted in Europe, while 3 were conducted in the United States. This study included 13 retrospective, 8 prospective, and 2 case-control studies. The studies conducted between 1978 and 2016 covered a wide range of years and identified *C. jejuni* and *C. coli* as the predominant pathogens causing campylobacteriosis. The study cohorts were selected from various sources, including 13 studies that focused on individuals who tested positive for *Campylobacter*, 4 studies obtained from population registries, 1 from a US military cohort, and 2 from foodborne outbreaks. The remaining three studies involved waterborne campylobacteriosis outbreaks. Age means, medians, or ranges were missing for 22% (5/23) of the studies. We evaluated the number of women who developed ReA in each study; however, this information has not been consistently reported. Of the 23 studies, 65% (15/23) did not report the number of women in their ReA cohorts. Additionally, we examined whether ReA patients were screened for HLA-B27 positivity. However, this information was unavailable in 65% (15/23) of the studies. In 70% (16/23) of the studies, campylobacteriosis infection was primarily confirmed through stool culture. The stool culture method was employed in 17% of the studies in combination with other approaches. In addition, enzyme immunoassays, laboratory-confirmed campylobacteriosis, and positive cultures were each reported in 4.3% of the studies. The diagnostic method for ReA was incorporated in all 23 studies. Among these studies, 43% (10/23) relied on self-reported ReA, 39% (9/23) used medical records, 9% (2/23) confirmed ReA diagnosis through specialists, and 9% (2/23) used a combination of diagnostic methods.

#### 3.1.1. Meta-Analysis of *Campylobacter* Studies

According to a review of 23 studies, the proportion of *Campylobacter* cases resulting in ReA was 0.03, with a 95% confidence interval (CI) of 0.01 to 0.06 (Figure 2).

Despite the study by Gumpel et al. (1981) being excluded [33], the overall proportion remained constant at 0.03, although this resulted in changes in the 95% confidence interval [0.01, 0.05].

The estimated variance between the true effects was high (τ^2^ = 2.93), indicating substantial heterogeneity between the studies. The proportion of total variation across studies due to heterogeneity (Higgin’s index) was found to be I^2^ = 97.62%, which confirms the significant heterogeneity attributed to between-study variation. Moreover, another measure of heterogeneity, the ratio of the variance of the true effect sizes to the sampling variance (H^2^ = 42.07), further confirmed the substantial heterogeneity between studies.

The results of Cochran’s Q test revealed significant heterogeneity among effect sizes across the included studies (Q(22) = 1055.91, *p* < 0.001). This suggests that the true effect sizes may vary substantially between studies, warranting further exploration into potential sources of heterogeneity.

The results of the meta-analysis revealed a significant overall effect size estimate (theta = 0, z = −9.57, *p* < 0.001). Based on the ReA diagnostic method, the subgroup meta-analysis results indicated no statistically significant differences between the subgroups (*p* = 0.12). This finding implies that the method used to assess ReA does not significantly influence the reported success rates.

#### 3.1.2. Assessment of Publication Bias

Funnel plot analysis revealed asymmetry, indicating a high likelihood of publication bias. However, the overall risk of bias assessment was low, as indicated in Figure S1.

Our analysis employed the trim-and-fill method to address potential publication bias in studies examining *Campylobacter* infection rates causing ReA development. Based on 23 included studies, the logit proportion was −3.546 (95% CI: [−4.272, −2.820]). After imputing potentially missing studies, the adjusted logit proportion was estimated to be −3.987 (95% CI: [−4.721, −3.252]), including 4 imputed studies. These findings suggest that publication bias may have influenced the original effect size estimate, and the trim and fill analysis provided a more accurate estimate by adjusting for potentially missing studies (Table S8).

#### 3.1.3. Cumulative Analysis

A cumulative meta-analysis forest plot was used to combine multiple studies to show the cumulative effect of the ReA proportion over time (Figure S2). The figure displays individual study effect estimates and their confidence intervals in chronological order from 1981 to 2022. As more studies are added, the effect estimates become more precise and stable, from 0.24 [0.13, 0.42] to 0.03 [0.01, 0.06], and the cumulative effect estimates show an overall proportion of 0.03 [0.01, 0.06]. Overall, the plot provides insights into the synthesized evidence of interventions or exposures over time.

**Table 1:** Studies of *Campylobacter*.

Source, First Author, Year	Country	Cohort Source	Species	Study Design	Study Duration	Mean Age (of Whole Cohort)	Subjects with *Campylobacter* Infection	Subjects Developed ReA	Proportion (%)/Occurrence	Campylobacteriosis Diagnosis	ReA Diagnosis	N of Women Developed ReA	N of HLA-B27 Positive
Bremell et al., 1991 [31]	Sweden	Foodborne outbreak	*C. jejuni*	Retrospective cohort	1986–1987	26.9	66	5	7.6	ELISA/stool culture	Self-report	4/5	1/5
Doorduyn et al., 2008 [32]	Netherlands	Population registry	Multiple	Case-control study	2002–2003	Median—60	434	20	4.6	Stool culture	Self-report	18/20	N/D
Gumpel et al., 1981 [33]	UK	Patients’ records	Multiple	Prospective cohort	1978	5–16; >16	33	8	24.2	Stool culture	Medical records	N/D	N/D
Eastmond et al., 1983 [34]	Scotland	Foodborne outbreak	*C. jejuni*	Prospective cohort	1979	N/D	130	2	0.8	Stool culture	Medical records	0/2	0/1
Hannu et al., 2002 [35]	Finland	Campylobacter-positive subjects	*C. coli*, *C. jejuni*	Case-control study	1997–1998	37.1	609	45	7.4	Stool culture	Confirmed by specialist	34/45	6/45
Hannu et al., 2004 [36]	Finland	Waterborne outbreak	*C. jejuni*	Prospective cohort	2000	58	350	9	2.6	Enzyme immunoassay	Confirmed by specialist	6/9	3/9
Helms et al., 2006 [37]	Denmark	Three National registries	Multiple	Retrospective cohort	1991–1999	Median—26	17,991	22	0.1	Stool culture	Medical records	N/D	N/D
Johnsen et al., 1983 [38]	Norway	Campylobacter-positive subjects	*C. jejuni*	Prospective cohort	1980–1981	N/D	37	5	13.5	Stool culture	Confirmed by specialist	1/5	0/5
Kosunen et al., 1981 [39]	Finland	Campylobacter-positive subjects	*C. jejuni*	Prospective cohort	1978–1979	N/D	342	8	2.3	Stool culture, agglutination test	Medical records	2/8	5/7
Locht and Krogfelt, 2002 [40]	Denmark	Campylobacter-positive subjects	*C. coli*, *C. jejuni*	Retrospective cohort	1997–1999	Median—36	173	27	15.6	Stool culture/ELISA	Self-report	17/27	N/D
Melby et al., 2000 [41]	Norway	Waterborne outbreak	*C. coli*, *C. jejuni*	Retrospective cohort	1988	40.9	330	2	0.6	Stool culture	Self-report	N/D	N/D
Petersen et al., 1996 [42]	Denmark	Bacterial gastroenteritis patients	*C. coli*, *C. jejuni*	Retrospective cohort	1991–1993	Median—33	41	0	0	Blood/stool culture	Medical records	0	N/D
Pitkänen et al., 1981 [43]	Finland	Diarrheal patients	*C. jejuni*	Retrospective cohort	1978–1980	11–76	55	3	5.4	Stool culture	Self-report	1/2	N/D
Pitkänen et al., 1983 [44]	Finland	Campylobacter-positive subjects	*C. jejuni*	Retrospective cohort	1978–1981	0–89	188	9	4.8	Stool culture	Medical records	N/D	N/D
Pönkä et al., 1984 [45]	Finland	Campylobacter-positive subjects	*C. jejuni*	Retrospective cohort	1978–1981	0–70+	283	6	2.1	Stool culture	Self-reported	N/D	N/D
Porter et al., 2013 [46]	USA	US military medical database	Multiple	Retrospective cohort	1998–2009	N/D	738	1	0.13	Positive culture	Medical records	N/D	N/D
Rees et al., 2004 [25]	USA	Active Surveillance Network	Multiple	Retrospective cohort	1998–1999	N/D	324	9	2.8	Laboratory-confirmed	Self-report	6/9	N/D
Schiellerup et al., 2008 [47]	Denmark	Campylobacter-positive subjects	Multiple	Prospective cohort	2002–2003	Median—40	1003	131	13.1	Stool culture	Self-report	N/D	12/91
Schönberg-Norio et al., 2010 [48]	Finland	Campylobacter-positive patients	*C. jejuni*	Prospective cohort	2002	Median—50	201	8	4	Stool culture	Medical records	4/8	N/D
Ternhag, 2008 [49]	Sweden	Patients with GI infections	Multiple	Retrospective cohort	1997–2007	37	57,425	15	0.02	Stool culture	Medical records	N/D	N/D
Townes et al., 2008 [6]	USA	Culture-confirmed infections	*C. coli*, *C. jejuni*	Prospective cohort	2002–2004	Median—35	2384	33	1.4	Stool culture	Confirmed by specialist	36/52	6/52
Walker et al., 2022 [50]	New Zealand	Waterborne outbreak	Multiple	Retrospective cohort	2016	Median—47	106	19	17.9	Stool culture	Self-report	8/19	N/D
Zia et al., 2003 [51]	UK	Culture-confirmed *C. jejuni* enteritis	*C. jejuni*	Retrospective cohort	1999	47.7	155	6	3.9	Stool culture	Self-report	N/D	N/D

### 3.2. Escherichia

**Figure 3:**
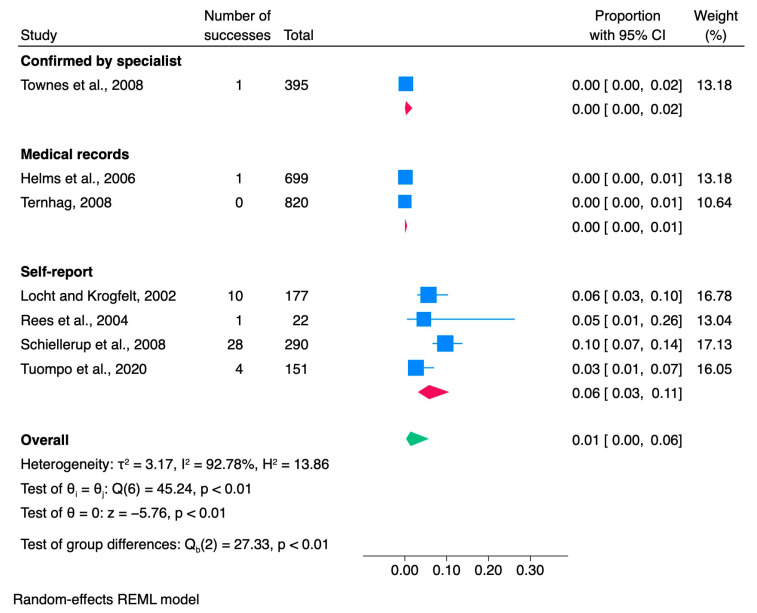
Subgroup meta-analysis of ReA cases among patients with *Escherichia* infection [6,25,37,40,47,49,53]. CI, confidence interval.

Seven articles were included in the meta-analysis to investigate the development of ReA triggered by diarrheagenic *Escherichia coli* infections (Table 2). DEC or diarrheagenic *E. coli* comprises five pathotypes, namely, enteropathogenic *E. coli* (EPEC), enteroaggregative *E. coli* (EAEC), enterotoxigenic *E. coli* (ETEC), enteroinvasive *E. coli* (EIEC), and enterohemorrhagic *E. coli* (EHEC) [52]. The meta-analysis included seven *E. coli* studies, which included 2554 cases of *Escherichia* infection. Among those patients, 45 patients developed ReA, resulting in an overall proportion of 0.01 [0.00, 0.06] (see Figure 3). Notably, two of the studies focused on pediatric patients. The studies were primarily conducted in Europe, with five out of seven occurring in this region. The other two studies were conducted in the United States. The methodologies utilized by these studies were diverse, with four retrospective designs and three prospective studies. The studies were conducted over a period of 19 years, from 1991 to 2010. The study cohorts were drawn from diverse sources, including three studies investigating *Escherichia*-positive patients, two studies utilizing data from population registries, one study involving patients with gastrointestinal infections, and another study involving a cohort of travel clinic volunteers. Age information, including means, medians, or ranges, was missing from 14% (1/7) of the studies. Of the seven studies, 43% (3/7) did not report the number of women in their ReA cohorts. We also checked whether ReA patients were screened for HLA-B27 positivity, but this information was not available for 71% (5/7) of the studies included in the analysis. In 71% (5/7) of the studies, stool culture was used as the primary method to confirm *Escherichia* infection. Additionally, qPCR and laboratory-confirmed infections were each reported in 14% (1/7) of the studies. All seven studies included the diagnostic method for ReA. Among these studies, 57% (4/7) relied on self-reported ReA, 29% (2/7) used medical records, and 14% (1/7) confirmed ReA diagnosis through specialists.

#### 3.2.1. Meta-Analysis of *Escherichia* Studies

According to the results of a meta-analysis, the proportion of ReA cases stemming from an *Escherichia* infection was 0.01, with a 95% CI ranging from 0.00 to 0.06, as shown in Figure 3. The analysis revealed a high estimated variance between the true effects (τ^2^ = 3.17), indicating significant heterogeneity across the studies. Higgins’s index, which measures the proportion of total variation across studies due to heterogeneity, was found to be I^2^ = 92.78%, further confirming the significant heterogeneity attributed to between-study variation. Another measure of heterogeneity, the ratio of the variance of the true effect sizes to the sampling variance (H^2^ = 13.86), also confirmed the substantial heterogeneity between studies. According to the results of Cochran’s Q test, there was significant heterogeneity among the effect sizes in the studies analyzed (Q(6) = 45.24, *p* < 0.001). This finding implies that there may be considerable variation in the actual effect sizes across the studies, necessitating a deeper exploration into the potential causes of this heterogeneity. The meta-analysis showed a significant overall effect size estimate (θ = 0, z = −5.76, *p* < 0.001).

The subgroup meta-analysis, conducted using the ReA diagnostic method, indicated a statistically significant difference between subgroups (Qb (2) = 27.33, *p* < 0.01). These findings strongly suggest that the choice of method for assessing the ReA significantly influences the reported success rate.

#### 3.2.2. Assessment of Publication Bias

The analysis of the funnel plot showed that there was asymmetry, indicating a high probability of publication bias. However, the overall assessment of the risk of bias was low, as demonstrated in Figure S1. Notably, the proportion of patients with reactive arthritis (ReA) significantly contributed to the observed heterogeneity.

Trim and fill analysis was conducted to address potential publication bias in studies examining the proportion of ReA after *Escherichia* bacterial infection. Based on seven included studies, the observed logit proportion was −4.269 (95% CI: [−5.723, −2.815]). After imputing one potentially missing study, the adjusted logit proportion was estimated at −3.907 (95% CI: [−5.416, −2.398]). The total number of studies included in the analysis was eight, with seven observed studies and one imputed study. This analysis revealed that the observed effect size estimate for ReA proportions may have been influenced by publication bias. By imputing one potentially missing study, the adjusted effect size estimate was slightly altered, suggesting a potential impact of publication bias on the original estimate (Table S9).

#### 3.2.3. Cumulative Analysis

A forest plot of the cumulative meta-analysis was generated to merge several studies and illustrate the lasting impact of the ReA proportion. The plot portrays the individual effect estimates of each study and their corresponding confidence intervals arranged by year from 2002 to 2020 (Figure S3). The cumulative effect estimates reveal an overall proportion of 0.01 [0.00, 0.06]. Nevertheless, the plot offers valuable insights into the combined evidence of interventions or exposures over time.

**Table 2:** Studies of *Escherichia*.

Source, First Author, Year	Country	Cohort Source	Species	Study Design	Study Duration	Mean Age (of Whole Cohort)	Subjects with *Escherichia* Infection	Subjects Developed ReA	Proportion (%)/Occurrence	*Escherichia* Diagnosis	ReA Diagnosis	N of Women Developed ReA	N of HLA-B27 Positive
Helms et al., 2006 [37]	Denmark	3 National registries	*DEC *^1^ *(EHEC *^2^, *ETEC *^3^, *EPEC *^4^, *EIEC *^5^)	Retrospective cohort	1991–1999	Median—1	699	1	0.14	Stool culture	Medical records	N/D	N/D
Locht and Krogfelt, 2002 [40]	Denmark	*E. coli*—positive subjects	*DEC *^1^ *(ETEC *^3^*)*	Retrospective cohort	1997–1999	Median—43	177	10	5.64	Stool culture	Self-report	5/10	N/D
Rees et al., 2004 [25]	United States	Active Surveillance Network	*DEC *^1^ *(STEC *^9^, *E. coli O157:H7)*	Retrospective cohort	1998–1999	N/D	22	1	4.5	Laboratory-confirmed	Self-report	1/1	N/D
Schiellerup et al., 2008 [47]	Denmark	*Escherichia*—positive subjects	*DEC *^1^ (*ETEC *^2^, *A/EEC *^7^, *EPEC *^4^, *VTEC *^8^)	Prospective cohort	2002–2003	Median—40	290	28	9.7	Stool culture	Self-report	N/D	2/20
Ternhag, 2008 [49]	Sweden	Patients with GI infections	*DEC *^1^*(EHEC *^2^)	Retrospective cohort	1997–2004	F—25, M—19	820	0	0	Stool culture	Medical records	N/D	N/D
Townes et al., 2008 [6]	United States	Culture-confirmed infections	*DEC *^1^ (*STEC *^9^, *E. coli O157*)	Prospective cohort	2002–2004	Median—15	395	1	0.2	Stool culture	Confirmed by specialist	26/52	N/D
Tuompo et al., 2020 [53]	Finland	Travel Clinic Volunteers	*DEC *^1^ (*EHEC *^2^, *ETEC *^3^, *EPEC *^4^, *EIEC *^5^, *EAEC *^6^)	Prospective cohort	2009–2010	39.5	151	4	2.6	qPCR	Self-report	3/4	0/4

^1^ DEC (diarrheagenic *Escherichia coli*) includes ^2^ EHEC (enterohaemorrhagic *Escherichia coli*), ^3^ ETEC (enterotoxigenic *Escherichia coli*), ^4^ EPEC (enteropathogenic *Escherichia coli*), ^5^ EIEC (enteroinvasive *Escherichia coli*), and ^6^ EAEC (enteroaggregative *Escherichia coli*). ^7^ A/EEC (attaching and effacing *Escherichia coli*) belongs to the EPEC pathotype. ^8^ VTEC (verocytotoxin-producing *Escherichia coli*) or, in the USA, ^9^ STEC (Shiga toxin-producing *Escherichia coli*) belongs to the EHEC pathotype. *E. coli O157:07* belongs to this pathotype.

### 3.3. Salmonella

This study aimed to evaluate the occurrence of reactive arthritis (ReA) following *Salmonella* infection. Thirty different articles were analyzed, including 70,965 confirmed or probable cases of *Salmonella* infection (as shown in Table 3). Of these individuals, 639 went on to develop ReA, resulting in an overall proportion of 0.04 (with a 95% confidence interval of [0.02, 0.08]). Most of the studies (17 out of 30) were conducted in Europe, 11 were conducted in North America (including Canada and the United States), and 2 were undertaken in Australia. Three studies also included participants under the age of 18. Most of the studies (18 out of 30) were retrospective, eight were prospective, and four were case-control studies. The studies covered a broad spectrum of periods, from 1953 to 2005. The most common pathogens associated with salmonellosis were *S. enteritidis* and *S. typhimurium*. The study cohorts were derived from diverse sources, including 8 studies that investigated Salmonella-positive patients, 3 that used data from population registries, 1 that utilized the US Department of Defense Medical Database, and 16 studies that were based on data from foodborne outbreaks. Additionally, two studies focused on salmonellosis outbreaks with no known source. In 27% (8/30) of the studies we reviewed, information regarding age, medians, or ranges was missing. We evaluated the number of women who developed ReA in each study, but these data were inconsistent. Among the 30 studies, 33% (10/30) did not specify the number of women in their ReA cohorts. Furthermore, we examined whether ReA patients were screened for HLA-B27 positivity, but this information was unavailable in 40% (12/30) of the studies we included. In 67% (20/30) of the studies, salmonellosis infection was confirmed primarily through stool culture, while in 17% (5/30) of the studies, the stool culture method was employed in combination with other diagnostic approaches. In 3% (1/30) of the studies, salmonellosis was confirmed by laboratory tests. Similarly, positive cultures were also reported in 3% (1/30) of the studies. ELISA/immunoblotting was also positive in 3% (1/30) of the studies. Moreover, in 6% (2/30) of the studies, the method of bacterial infection diagnosis was not specified. All 30 studies included in the research reported the diagnostic method for ReA. Of these studies, 12 out of 30 (40%) relied on self-reported ReA, 8 out of 30 (27%) used medical records, 8 out of 30 (27%) confirmed the ReA diagnosis through specialists, and 2 out of 30 (6%) used a combination of diagnostic methods.

#### 3.3.1. Meta-Analysis of *Salmonella* Studies

**Figure 4:**
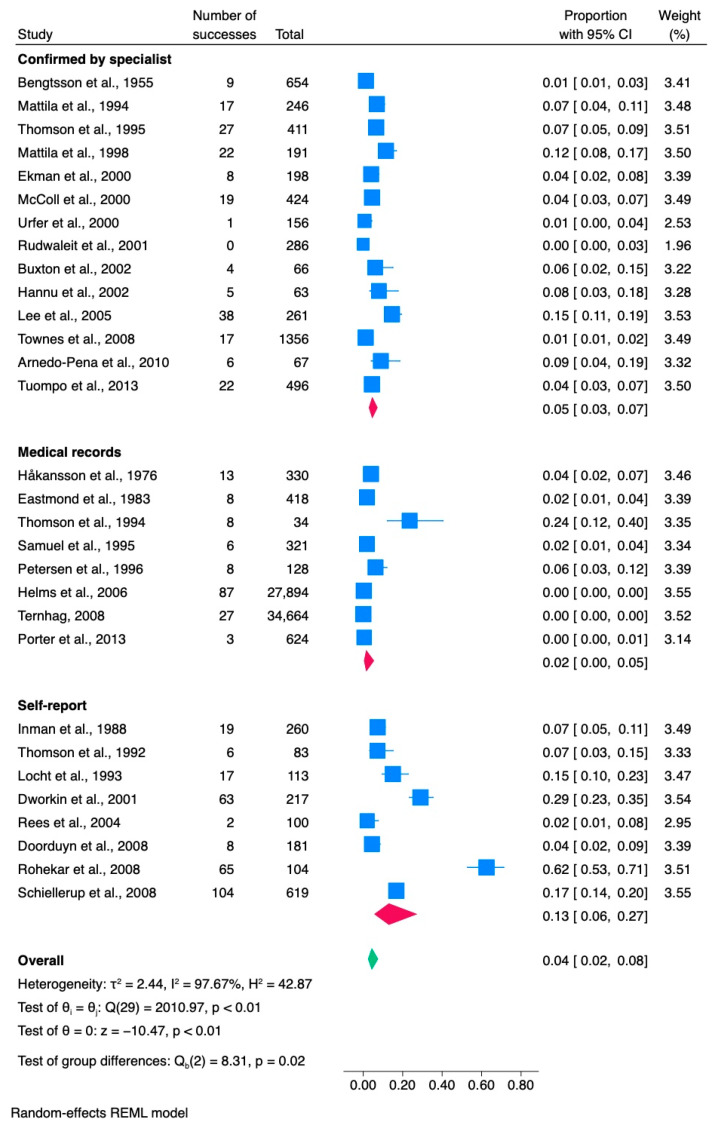
Subgroup meta-analysis of ReA cases among patients with *Salmonella* infection [6,25,32,33,35,37,42,46,47,49,54,55,56,57,58,59,60,61,62,63,64,65,66,67,68,69,70,71,72,73]. CI, confidence interval.

A review of 30 studies revealed that the proportion of *Salmonella* cases resulting in ReA was 0.04, with a 95% CI [0.02, 0.08], as shown in Figure 4. After excluding the study by Rohekar et al. (2008) [54], the overall proportion remained unchanged. However, the 95% CI became narrower [0.02, 0.06].

The estimated variance between the true effects was high (τ^2^ = 2.44), indicating substantial heterogeneity between the studies. The proportion of total variation across studies due to heterogeneity (Higgin’s index) was found to be I^2^ = 97.67%, which confirms the significant heterogeneity attributed to between-study variation. Additionally, another measure of heterogeneity, the ratio of the variance of the true effect sizes to the sampling variance (H^2^ = 42.87), further confirmed the substantial heterogeneity between studies. The results of Cochran’s Q test revealed significant heterogeneity among effect sizes across the included studies (Q(29) = 2010.97, *p* < 0.001). This suggests that the true effect sizes may vary substantially between studies, warranting further exploration into potential sources of heterogeneity. The results of the meta-analysis revealed a significant overall effect size estimate (theta = 0, z = −10.47, *p* < 0.001).

The results of the subgroup meta-analysis according to the ReA diagnostic method revealed a statistically significant difference between the subgroups (*p* = 0.02). This suggests that the assessment method for ReA has a notable impact on the reported success rates.

#### 3.3.2. Assessment of Publication Bias

Funnel plot analysis revealed asymmetry, indicating a high likelihood of publication bias. However, the overall risk of bias assessment was low, as indicated in Figure S1. Notably, the proportion of ReA significantly contributed to the observed heterogeneity.

Trim and fill analysis was conducted to check for potential publication bias in studies on *Salmonella* infection rates. The observed logit proportion, based on 30 included studies, was found to be −3.090 (95% CI: [−3.669, −2.512]). No additional studies were added, resulting in an unchanged adjusted logit proportion of −3.090 (95% CI: [−3.669, −2.512]). All 30 studies were observed, and none needed to be imputed. These results indicate that there was no evidence of publication bias affecting the estimated *Salmonella* infection rates among the included studies. The lack of imputed studies suggests that the observed effect size estimate remained consistent, reinforcing the robustness of the findings (Table S10).

#### 3.3.3. Cumulative Analysis

A cumulative meta-analysis forest plot was used to combine multiple studies to show the cumulative effect of the ReA proportion over time (Figure S4). Individual study effect estimates and their confidence intervals are displayed in chronological order from 1955 to 2013. The cumulative effect estimates showed an overall proportion of 0.04 [0.02, 0.08]. Overall, the plot provides insights into the synthesized evidence of interventions or exposures over time.

**Table 3:** Studies of *Salmonella*.

Source, First Author, Year	Country	Cohort Source	Species	Study Design	Study Duration	Mean Age (of Whole Cohort)	Subjects with *Salmonella* Infection	Subjects Developed ReA	Proportion (%)/Occurrence	Salmonellosis Diagnosis	ReA Diagnosis	N of Women Developed ReA	N of HLA-B27 Positive
Arnedo-Pena et al., 2010 [55]	Spain	Foodborne outbreak	*S. hadar*	Prospective cohort	2005	34.5	67	6	9	Stool culture	Confirmed by specialist	1/6	N/D
Bengtsson et al., 1955 [56]	Sweden	Foodborne outbreak	*S. typhimurium*	Retrospective cohort	1953–1955	N/D	654	9	1.3	Stool culture, agglutination test	Confirmed by specialist	3/9	N/D
Buxton et al., 2002 [57]	Canada	*S. Typhimurium*—confirmed cases	*S. typhimurium*	Case-control study	1999–2000	N/D	66	4	6	Stool culture	Confirmed by specialist	1/4	N/D
Doorduyn et al., 2008 [32]	Netherlands	Population registry	*S. enteritidis*, *S. typhimurium*	Case-control study	2002–2003	N/D	181	8	4.4	Stool culture	Self-report	7/8	N/D
Dworkin et al., 2001 [58]	United States	Foodborne outbreak	*S. enteritidis*	Retrospective cohort	1994	Median—35	217	63	29	Diarrhea	Self-report	N/D	N/D
Eastmond et al., 1983 [33]	Scotland	Foodborne outbreak	*S. typhimurium*	Prospective cohort	1981	N/D	418	8	1.9	Stool culture	Medical records	3/8	3/8
Ekman et al., 2000 [59]	Finland	*Salmonella*—infected patients	*S. enteritidis*, *S. hadar*, *S. typhimurium*, *S. infantis*, *S. stanley*	Prospective cohort	1998–1999	39.1	198	8	4	Stool culture	Confirmed by specialist	4/8	6/8
Håkansson et al., 1976 [60]	Sweden	Salmonellosis outbreak	*S. typhimurium*	Retrospective cohort	1974	Range 17–61	330	13	3.9	N/D	Medical records	4/13	9/13
Hannu et al., 2002 [35]	Finland	Salmonellosis outbreak	*S. typhimurium*	Retrospective cohort	1999	Median—30.7	63	5	7.9	Stool culture	Confirmed by specialist	3/5	2/4
Helms et al., 2006 [37]	Denmark	3 National registries	*S. enteritidis*, *S. typhimurium*	Retrospective cohort	1991–1999	Median—34	27,894	87	0.3	Stool culture	Medical records	N/D	N/D
Inman et al., 1988 [61]	Canada	Foodborne outbreak	*S. typhimurium*	Retrospective cohort	1984	39.3	260	19	7.3	Stool culture	Self-report	0/19	4/11
Lee et al., 2005 [62]	Australia	Foodborne outbreak	*S. typhimurium*	Retrospective cohort	1999–2001	15	261	38	14.6	Stool culture	Confirmed by specialist	N/D	5/30
Locht et al., 1993 [63]	Sweden	Foodborne outbreak	*S. enteritidis*	Retrospective cohort	1990	F—49.4, M—52.6	113	17	15	Stool culture	Self-report	8/17	N/D
Mattila et al., 1994 [64]	Finland	Foodborne outbreak	*S. enterica*	Prospective cohort	1992	Median—14	246	17	6.9	Stool culture, enzyme immunoassay	Confirmed by specialist	15/17	4/13
Mattila et al., 1998 [65]	Finland	Foodborne outbreak	*S. bovismorbificans*	Retrospective cohort	1994	Median—32	191	22	11.5	Stool culture, enzyme immunoassay	Confirmed by specialist	15/22	10/22
McColl et al., 2000 [66]	Australia	Foodborne outbreak	*S. typhimurium*	Retrospective cohort	1997	28	424	19	4.5	Stool culture	Confirmed by specialist	11/19	2/19
Petersen et al., 1996 [42]	Denmark	Bacterial gastroenteritis patients	*S. paratyphi*, *S. typhi*, *S. enteriditis*	Retrospective cohort	1991–1993	Median—33	128	8	5.9	Blood/stool culture	Medical records	N/D	N/D
Porter et al., 2013 [46]	United States	US Department of Defense medical database	Multiple	Retrospective cohort	1998–2009	N/D	624	3	0.5	Positive culture	Medical records	N/D	N/D
Rees et al., 2004 [25]	United States	Active Surveillance Network	Multiple	Retrospective cohort	1998–1999	N/D	100	2	2	Laboratory-confirmed	Self-report	0/2	N/D
Rohekar et al., 2008 [54]	Canada	Foodborne outbreak	*S. enteriditis*	Prospective cohort	2005	46	104	65	62.5	Stool culture	Self-report	N/D	5/37
Rudwaleit et al., 2001 [67]	Germany	*Salmonella*-positive subjects	*S. enteriditis*	Retrospective cohort	1998	Range (11 months—9 years)	286	0	0	Stool culture	Confirmed by specialist	0	0
Samuel et al., 1995 [68]	United States	Foodborne outbreak	*S. typhimurium*	Retrospective cohort	1993	N/D	321	6	1.8	Stool culture	Medical records	N/D	3/5
Schiellerup et al., 2008 [47]	Denmark	*Salmonella*-positive subjects	*S. typhimurium*, *S. enteritidis*; other	Prospective cohort	2002–2003	Median—40	619	104	16.8	Stool culture	Self-report	N/D	19/86
Ternhag, 2008 [49]	Sweden	Patients with GI infections	Nontyphoidal *Salmonella* spp. (*S. enteritidis*, *S. typhimurium*, *S. virchow*, *S. hadar*, and others)	Retrospective cohort	1997–2004	F—37, M—36	34,664	27	0.08	Stool culture	Medical records	N/D	N/D
Thomson et al., 1992 [69]	Canada	Foodborne outbreak	*S. heidelberg*, *S. hadar*, *S. thomson*	Case–control study	1992	40.6	83	6	7.2	Immunoblotting/ELISA	Self-report	5/6	0/6
Thomson et al., 1994 [70]	Canada	Foodborne outbreak	*S. enteritidis*	Case–control study	1990–1992	N/D	29	8	27.5	Stool culture/ELISA	Medical records	3/8	3/8
Thomson et al., 1995 [71]		Foodborne outbreak	*S. typhimurium*	Retrospective cohort	1989	39	411	27	6.3	Stool culture	Confirmed by specialist	N/D	6/27
Townes et al., 2008 [6]	United States	Culture-confirmed infections	*S. typhimurium*, *S. enteritidis*, *S. newport*, *S. Heidelberg*, and others	Prospective cohort	2002–2004	Median—29	1356	17	1.25	Stool culture	Confirmed by specialist	N/D	N/D
Tuompo et al., 2013 [72]	Finland	Salmonella-positive subjects	*S. typhimurium*, *S. enteritidis*, *S. paratyphi*, and others	Prospective cohort	2003–2005	40.5	496	22	4.4	Stool culture	Confirmed by specialist	9/22	5/12
Urfer et al., 2000 [73]	Switzerland	Foodborne outbreak	*S. braenderup*	Retrospective cohort	1993–1994	Median—32	156	1	0.6	Stool culture	Confirmed by specialist	1/1	N/D

### 3.4. Shigella

**Figure 5:**
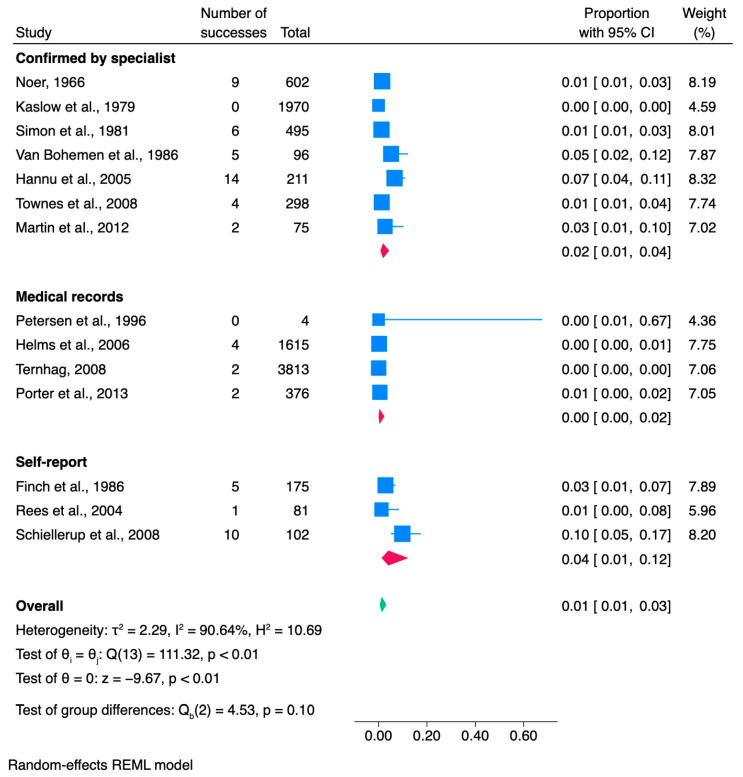
Subgroup meta-analysis of ReA cases among patients with *Shigella* infection [6,25,37,42,46,47,49,74,75,76,77,78,79,80]. CI, confidence interval.

A comprehensive review of 14 research papers was conducted to determine the correlation between the prevalence of ReA and *Shigella* infection (Table 4). The collective data encompassed 9913 confirmed or probable cases of shigellosis, from which 64 cases of ReA emerged. The analysis indicated that the proportion of ReA associated with *Shigella* infection was 0.01, with a 95% confidence interval ranging from 0.01 to 0.03, as illustrated in Figure 5. One of the studies included individuals under 18 years of age. Geographically, the research was distributed across six studies in Europe, six in the United States, one in Puerto Rico, and one in Afghanistan. Seven of the fourteen studies were retrospective, six were prospective, and one was a case-control study. These studies included data from 1962 to 2011, representing various periods. The primary pathogens associated with shigellosis were identified as *S. sonnei* and *S. flexneri*. The study cohorts were gathered from diverse sources: Five studies analyzed data from foodborne outbreaks, three studies focused on *Shigella*-positive patients, two studies utilized data from population registries, one study examined a shigellosis outbreak with an unidentified source, and another used the US Department of Defense medical database. In 50% of the studies (7/14), age, medians, or ranges were not reported.

Similarly, the number of women who developed ReA in each study was not consistent, with 36% (5/14) of the studies not including this information. Additionally, 42% (6/14) of the studies did not report whether ReA patients were screened for HLA-B27 positivity. Shigellosis was primarily confirmed through stool culture in 11 of 14 studies (80%). The laboratory method used to confirm *Shigella* infection was not specified in the remaining three studies (20%). All 14 studies included a diagnostic method for ReA. Among these studies, three (23%) relied on self-reported ReA, four (28%) used medical records, six (42%) confirmed ReA diagnosis through specialists, and one (7%) used a combination of diagnostic methods.

#### 3.4.1. Meta-Analysis of *Shigella* Studies

A meta-analysis of 14 studies revealed that the proportion of *Shigella* cases resulting in ReA was 0.01 (95% CI [0.01, 0.03]), as illustrated in Figure 5.

The estimated variance between the true effects was high (τ^2^ = 2.29), indicating significant heterogeneity between the studies. The proportion of total variation across studies due to heterogeneity (Higgins index) was I^2^ = 90.64%, confirming substantial heterogeneity attributed to between-study variation. Another measure of heterogeneity, the ratio of the variance of the true effect sizes to the sampling variance (H^2^ = 10.69), further confirmed significant heterogeneity between studies. The Cochran’s Q test revealed significant variations in effect sizes among the studies analyzed (Q(13) = 111.32, *p* < 0.01), suggesting that the true effect sizes could differ considerably across studies. This underscores the importance of exploring potential sources of variation in greater depth. The analysis results revealed a substantial overall effect size estimate (theta = 0, z = −9.67, *p* < 0.01). These results suggest that there is a true effect present in the studies included in the analysis. The subgroup meta-analysis results suggested that there were no statistically significant differences between subgroups (*p* = 0.10) based on the ReA diagnostic method. This indicates that the method used to assess ReA does not significantly influence the reported success rates.

#### 3.4.2. Assessment of Publication Bias

Funnel plot analysis revealed asymmetry, indicating potential publication bias. However, the overall assessment of bias risk was low, as depicted in Figure S1.

Our analysis utilized the trim-and-fill method to address potential publication bias within studies investigating the proportion of ReA cases after *Shigella* infection. The observed logit proportion, derived from 14 included studies, was −4.292 (95% CI: [−5.162, −3.422]). After accounting for potentially missing studies, the adjusted logit proportion remained at −4.292 (95% CI: [−5.162, −3.422]), with no additional studies imputed. This suggests that publication bias likely had minimal influence on the original effect size estimate, and the trim and fill analysis did not alter the initial estimation (Table S11).

#### 3.4.3. Cumulative Analysis

The forest plot represents a cumulative meta-analysis showing the combined effect of the ReA proportion across multiple studies. Studies are arranged chronologically from earliest to most recent, spanning from 1966 to 2013. With the inclusion of more studies over time, there is a trend toward increased precision in the effect estimates, transitioning from wider to narrower confidence intervals. For instance, the proportion estimate ranges from 0.02 with a wider 95% CI [0.01, 0.05] in earlier studies to 0.01 with a narrower CI [0.01, 0.03] in later studies. The cumulative effect estimates converge toward an overall proportion of 0.01 [0.01, 0.03].

**Table 4:** Studies of *Shigella*.

Source, First Author, Year	Country	Cohort Source	Species	Study Design	Study Duration	Mean Age (of Whole Cohort)	Subjects with *Shigella* Infection	Subjects Developed ReA	Proportion (%)/Occurrence	Shigellosis Diagnosis	ReA Diagnosis	N of Women Developed ReA	N of HLA-B27 Positive
Finch et al., 1986 [74]	United States	Foodborne outbreak	*S. flexneri*	Prospective cohort	1982	N/D	175	5	2.8	Stool culture	Self-report	2/5	4/5
Hannu et al., 2005 [75]	Finland	*Shigella*—positive subjects	*S. sonnei*, *S. flexneri*, *S. dysenteriae*, *S. boydii*	Case—control study	1996–2000	37.8	211	14	6.6	Stool culture	Self-report/Confirmed by specialist	9/14	5/14
Helms et al., 2006 [37]	Denmark	3 National registries	Multiple	Retrospective cohort	1991–1999	Median—29	1615	4	0.24	Stool culture	Medical records	N/D	N/D
Kaslow et al., 1979 [76]	Puerto Rico	Large outbreak in local community	*S. sonnei*	Prospective cohort	1979	N/D	1970	0	0	Stool culture	Confirmed by specialist	0	0
Martin et al., 2012 [77]	Afghanistan	Foodborne outbreak	Multiple	Prospective cohort	2011	N/D	75	2	2.66	Stool culture	Confirmed by specialist	0/2	1/2
Noer, 1966 [78]	United States	Foodborne outbreak	Multiple	Retrospective cohort	1962	N/D	602	9	1.5	Stool culture	Confirmed by specialist	0/9	N/D
Petersen et al., 1996 [42]	Denmark	Bacterial gastroenteritis patients	*S. sonnei*	Retrospective cohort	1991–1993	Median—33	4	0	0	Blood/stool culture	Medical records	N/D	N/D
Porter et al., 2013 [46]	United States	US Department of Defense medical database	Multiple	Retrospective cohort	1998–2009	N/D	376	2	0.5	Positive culture	Medical records	N/D	N/D
Rees et al., 2004 [25]	United States	Active Surveillance Network	Multiple	Retrospective cohort	1998–1999	N/D	81	1	1.2	Laboratory-confirmed	Self-report	1/1	N/D
Schiellerup et al., 2008 [47]	Denmark	*Shigella*—positive subjects	*S. sonnei*, *S. flexneri*, other	Prospective cohort	2002–2003	Median—40	102	10	9.8	Stool culture	Self-report	(M/F 36.4%/63.6%)	2/6
Simon et al., 1981 [79]	United States	3 foodborne outbreaks	*S. sonnei*, *S. flexneri*	Prospective cohort	1978	N/D	495	6	1.2	Stool culture	Confirmed by specialist	6/6	5/6
Ternhag, 2008 [49]	Sweden	Patients with GI infections	Multiple	Retrospective cohort	1997–2004	33	3813	2	0.05	Stool culture	Medical records	N/D	N/D
Townes et al., 2008 [6]	United States	Culture-confirmed infections	*S. sonnei*, *S. flexneri*, *S. boydii*, *S. dysenteriae*	Prospective cohort	2002–2004	Median—21	298	4	1.3	Stool culture	Confirmed by specialist	N/D	N/D
van Bohemen et al., 1986 [80]	The Netherlands	Foodborne outbreak	*S. flexneri*	Retrospective cohort	1985	Range 10–100	96	5	5.2	Stool culture	Confirmed by specialist	N/D	5/5

### 3.5. Yersinia

**Figure 6:**
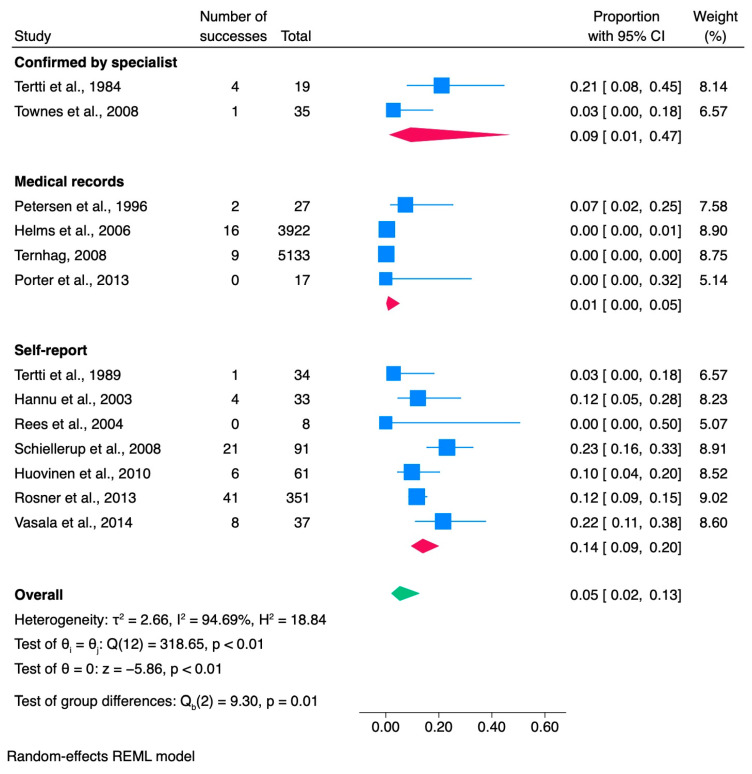
Subgroup meta-analysis of ReA cases among patients with *Yersinia* infection [6,25,37,42,46,47,49,81,82,83,84,85,86]. CI, confidence interval.

Thirteen articles were reviewed to evaluate the occurrence of ReA triggered by *Yersinia* infection (Table 5). These studies included 9768 patients with yersiniosis, 113 of whom subsequently developed ReA, resulting in an overall proportion of 0.05 (95% CI [0.02, 0.13]) (Figure 6). Two of the studies focused on pediatric patients. The majority of the studies were conducted in Europe (n = 10), with 5 out of the 10 studies originating from Finland. Additionally, three studies were conducted in the United States. The methodological approach of these studies varied, with the majority being retrospective (nine studies), while two were prospective, and two were case-control studies. The study data spanned the years from 1987 to 2010.

*Y. enterocolitica* was the predominant pathogen associated with yersiniosis, followed by *Y. pseudotuberculosis*. The study cohorts were drawn from diverse sources, including four studies based on data from foodborne outbreaks, one study from a yersiniosis outbreak of unknown etiology, three studies investigating *Yersinia*-positive patients, two studies utilizing data from population registries, and one study using the US Department of Defense medical database. Furthermore, two additional studies involved patients with gastrointestinal infections.

Age, medians, or ranges were not reported in 15% of the studies (2/13). The documentation of the incidence of ReA in women was inconsistent across the studies we assessed. Specifically, 55% (7/13) did not provide information on the number of women in their ReA cohorts. Additionally, 40% (5/13) of the studies lacked information on whether ReA patients were screened for HLA-B27 positivity. In 77% (10/13) of the studies, yersiniosis infection was predominantly confirmed through stool culture, while the remaining 23% (3/13) reported laboratory-confirmed infection and positive culture. The diagnostic approach for the assessment of ReA was specified in all 13 studies, with 54% relying on self-reported ReA, 30% relying on medical records, and 16% confirming ReA diagnosis through specialists.

#### 3.5.1. Meta-Analysis of *Yersinia* Studies

According to a comprehensive review of 13 studies, the proportion of Yersinia cases resulting in ReA was 0.05, with a 95% confidence interval of [0.02, 0.13] (Figure 6). When the study by Schiellerup et al. (2008) [47] was excluded, the overall proportion remained at 0.05, but the 95% confidence interval narrowed to [0.02, 0.11].

The results showed a high estimated variance between true effects (τ^2^ = 2.66), indicating significant heterogeneity among the studies. The Higgins index, which measures the proportion of total variation across studies due to heterogeneity, was determined to be I^2^ = 94.66%, confirming substantial heterogeneity attributed to between-study variation. Additionally, another measure of heterogeneity, the ratio of the variance of the true effect sizes to the sampling variance (H^2^ = 18.84), further supported the substantial heterogeneity between studies. The findings of Cochran’s Q test indicated significant heterogeneity among effect sizes across the included studies (Q(22) = 318.65, *p* < 0.01). This suggests that there is considerable variation in true effect sizes among the studies, underscoring the need for further investigation into potential sources of heterogeneity.

The results of the meta-analysis revealed a statistically significant overall effect size estimate (theta = 0, Z = −5.86, *p* < 0.01), indicating that a true effect was present in the studies analyzed, with the effect size significantly differing from zero. Additionally, the subgroup meta-analysis based on the ReA diagnostic method showed a statistically significant variance between subgroups (*p* = 0.01), highlighting the impact of the method used to assess ReA on the reported success rates.

#### 3.5.2. Assessment of Publication Bias

The analysis of the funnel plot showed asymmetry, suggesting a strong possibility of publication bias. Nevertheless, the overall assessment of bias risk was low, as shown in Figure S1.

The trim-and-fill method was utilized to account for potential publication bias in studies examining the proportion of ReA following *Yersinia* infection. The observed logit proportion, based on 13 included studies, was −2.884 (95% CI: [−3.849, −1.918]). After adjusting for potentially missing studies, the estimated logit proportion was −3.230 (95% CI: [−4.161, −2.299]), including two imputed studies. These findings suggest that publication bias may have influenced the original effect size estimate. The trim and fill analysis provided a more accurate estimate by addressing potentially missing studies (Table S12).

#### 3.5.3. Cumulative Analysis

A cumulative meta-analysis forest plot was used to combine multiple studies over time to show the cumulative effect of the ReA proportion over time (Figure S6). The figure displays individual study effect estimates and their confidence intervals in chronological order from 1984 to 2014. The proportion estimate started from 0.21 [0.08, 0.45] in earlier studies and reached 0.05 [0.02, 0.13] in later studies. The cumulative effect estimates converge toward an overall proportion of 0.05 [0.02, 0.13].

**Table 5:** Studies of *Yersinia*.

Source, First Author, Year	Country	Cohort Source	Species	Study Design	Study Duration	Mean Age (of Whole Cohort)	Subjects with *Yersinia* Infection	Subjects Developed ReA	Proportion (%)/Occurrence	Yersiniosis Diagnosis	ReA Diagnosis	N of Women Developed ReA	N of HLA-B27 Positive
Hannu et al., 2003 [81]	Finland	Foodborne outbreak	*Y. pseudotuberculosis*	Retrospective cohort	1998	24.7	33	4	12.1	Stool culture	Self-report	2/4	3/3
Helms et al., 2006 [37]	Denmark	3 National registries	*Y. enterocolitica*	Retrospective cohort	1991–1999	Median—4.3	3922	16	0.4	Stool culture	Medical records	N/D	N/D
Huovinen et al., 2010 [82]	Finland	*Yersinia*—positive subjects	*Y. enterocolitica*	Case—control study	2006	32	61	6	9.8	Stool culture	Self-report	N/D	N/D
Petersen et al., 1996 [42]	Denmark	Bacterial gastroenteritis patients	*Y. enterocolitica*	Retrospective cohort	1991–1993	Median—33	27	2	7.4	Blood/stool culture	Medical records	N/D	N/D
Porter et al., 2013 [46]	United States	US Department of Defense medical database	*Y. enterocolitica*	Retrospective cohort	1998–2009	N/D	17	0	0	Positive culture	Medical records	0	0
Rees et al., 2004 [25]	United States	Active Surveillance Network	Multiple	Retrospective cohort	1998–1999	N/D	8	0	0	Laboratory-confirmed	Self-report	0	0
Rosner et al., 2013 [83]	Germany	Foodborne outbreak	*Y. enterocolitica*	Case—control study	2009–2010	8	351	41	11.7	Stool culture	Self-report	N/D	N/D
Schiellerup et al., 2008 [47]	Denmark	*Yersinia*—positive subjects	*Y. enterocolitica*, other	Prospective cohort	2002–2003	Median—40	91	21	23	Stool culture	Self-report	N/D	4/18
Ternhag, 2008 [49]	Sweden	Patients with GI infections	*Y. enterocolitica*	Retrospective cohort	1997–2004	F—28, M—27	5133	9	0.2	Stool culture	Medical records	N/D	N/D
Tertti et al., 1984 [84]	Finland	Unknown etiology outbreak	*Y. pseudotuberculosis*	Retrospective cohort	1981–1982	23	19	4	21	Stool culture	Confirmed by specialist	1/4	3/4
Tertti et al., 1989 [85]	Finland	Foodborne outbreak	*Y. pseudotuberculosis*	Retrospective cohort	1987–1988	9	34	1	2.9	Stool culture	Self-report	0/1	1/1
Townes et al., 2008 [6]	United States	Culture-confirmed infections	Multiple	Prospective cohort	2002–2004	Median—31	35	1	2.8	Stool culture	Confirmed by specialist	N/D	N/D
Vasala et al., 2014 [86]	Finland	Foodborne outbreak	*Y. pseudotuberculosis*	Retrospective cohort	2008	Median—49	37	8	21.6	Stool culture	Self-report	N/D	6/9

## 4. Discussion

This study utilized a systematic literature search and meta-analysis to determine the percentage of patients who developed ReA among individuals with *Campylobacter*, *Escherichia*, *Salmonella*, *Shigella*, or *Yersinia* infections. The analysis primarily included retrospective cohort studies, which have the potential to introduce selection bias. Importantly, while the study design does not inherently introduce confounding effects, controlling for confounding factors is crucial in analyzing observational studies. The inclusion of retrospective studies may heighten the risk of selection bias, necessitating careful consideration during data analysis. Managing these factors within the studies is indeed challenging and requires meticulous control of potential confounders during the analysis phase. Retrospective studies rely on historical data and medical records, which may not always be complete or accurate [87].

The scarcity of studies on ReA may reflect underlying challenges related to the underreporting and underdiagnosis of enteric bacterial infections. Underreporting and underdiagnosis are intrinsic issues that occur independently in clinical practice, potentially leading to lower disease prevalence estimates in research settings. This lack of identification could lead to a failure to assess the connection between infection and ReA development in unrecognized patients. Furthermore, in instances where ReA occurrences were not documented, it remains unclear whether they were not assessed or if they were assessed but went unnoticed among the cases. To address this issue, it is essential to focus on improving the identification and recognition of the connection between infection and ReA development.

This study revealed exclusive bacterial infections without overlapping cases across the included studies. This finding may be attributed to several factors inherent to our study design and scope. Our systematic review focused on individual bacterial pathogens (*Campylobacter*, *Escherichia*, *Salmonella*, *Shigella*, and *Yersinia* spp.*)* and specifically excluded studies that reported overlapping infections or mixed etiologies. This deliberate approach aimed to provide a focused analysis of the incidence of ReA associated with each distinct pathogen. The exclusivity of infections observed in the study underscores the importance of considering study design and selection criteria when interpreting epidemiological data. Although this approach allowed for a detailed examination of ReA associated with specific pathogens, it may limit the generalizability of our findings to settings where overlapping infections are more prevalent. Future studies could explore the prevalence of overlapping infections and their clinical implications to further enhance our understanding of the epidemiology of ReA. Additionally, considering broader inclusion criteria in future meta-analyses may provide insights into the complex interactions between multiple bacterial pathogens and ReA.

In the systematic review and meta-analysis, the number of reports on discrete bacterial infections exhibited significant variability. This variability in the number of reports may be attributable to a range of contributing factors. First, ReA lacks a specific diagnostic test and relies solely on clinical characteristics and is thus dependent on clinical judgment. Furthermore, no established criteria for ReA diagnosis exist, leading to a broad spectrum of symptoms and definitions [88]. For instance, inconsistent definitions for probable *Campylobacter* cases across studies may influence the accuracy of reported proportions. Studies with broader definitions will have higher case numbers and lower sequela estimates than those with more rigorous definitions [89]. Second, currently, there are no definitive recommendations for routine testing to identify the causative bacterium of reactive arthritis [90]. Third, not having a definite time period from infection to ReA onset introduces considerable uncertainty and discrepancies among studies, leading to varying reactive arthritis incidence estimates [88]. In other words, there are several factors influencing the observed variability.

To assess whether this variability aligns with the incidence ratios of ReA and to what extent, the incidence of ReA triggered by different bacterial agents must be considered. For instance, it has been reported that approximately 50% of cases of ReA are caused by pathogens such as *Chlamydia trachomatis*, *Yersinia*, and *Salmonella* [90,91]. With this in mind, it would be reasonable to conclude that these infections might have greater incidences of ReA than other bacterial agents and might contribute more to the observed variability in reports than other bacterial organisms. Nevertheless, we acknowledge that drawing definite conclusions regarding the alignment of variability with arthritis incidence ratios is still complex due to variability in identifying bacteria or bacterial products in the joint among different triggering agents, and comprehensive investigations are not always performed in every case [91]. Thus, in future research, comprehensive statistical analysis is needed to compare the observed ReA incidence ratios to the expected ratios for each bacterial agent. Such analysis would provide more clarity regarding this alignment.

Various subgroup meta-analyses were conducted to group the studies based on how the bacterial infection was identified (immunological methods, microbiological methods, or combined methods), the sources of the cohorts (bacterium-infected patients, outbreaks, or registries), geographical regions (Europe, North America, New Zealand, Middle East), bacterial species, and study designs (case-control studies, prospective and retrospective studies). However, grouping based on how ReA was diagnosed showed the most statistically significant differences between groups, as previously demonstrated by Pogreba-Brown et al. (2021) [26]. It is highly unlikely that the observed heterogeneity between subgroups was due to chance alone. We attempted to conduct a meta-regression analysis; however, for reasons such as an insufficient number of studies or substantial missing data on predictors, the findings did not demonstrate statistical significance. Consequently, we opted not to include the meta-regression analysis in the article. Although the present outcome may be deemed unsatisfactory, it presents a valuable opportunity for future research to examine this aspect in greater depth or consider alternative approaches to uncover any potential associations that may have eluded in this analysis.

The heterogeneity within and between studies for each bacteria investigated was greater than 90%. This could be attributed to varying study designs, including prospective, retrospective, and case-control studies, as well as the inclusion of children in some articles. It is worth noting that children may present with distinct disease manifestations, responses to treatment, and outcomes compared to adults. Consequently, this introduces some variability in the data.

Additionally, the use of different methods for ReA diagnosis, such as self-reported cases through phone interviews or mailed questionnaires (43% after campylobacteriosis, 57% after *Escherichia* infection, 40% after salmonellosis, and 55% after yersiniosis), may have contributed to the high level of heterogeneity. Relying on self-reported data from different sources not only introduces potential recall and reporting biases but also limits the standardized precision of clinical diagnoses.

The percentage of self-reported ReA was particularly high for four out of five bacteria investigated, with the lowest percentage of confirmed cases by medical specialists, with rates of 9% after campylobacteriosis, 14% after *Escherichia* infection, 27% after salmonellosis, and 15% after yersiniosis. The high percentage of self-reported ReA cases compared to confirmed cases by medical specialists across the investigated bacteria raises critical questions about the reliability and accuracy of the reported data.

One of the strengths of this systematic review and meta-analysis is the comprehensive and systematic literature search, which enables the capture of a broader spectrum of evidence. Additionally, this study addressed publication bias. Addressing publication bias provides a more accurate representation of the available evidence, reducing the risk of drawing biased conclusions.

Despite its strengths, this systematic review and meta-analysis has limitations. First, one of the primary limitations is the presence of significant heterogeneity among the studies included. Reports on HLA-B27 positivity, the number of female participants, and age statistics (mean, median, and range) were inconsistent across the studies. This lack of consistency made it challenging to perform a comprehensive statistical analysis and incorporate all relevant variables into the research. Another limitation relates to the variability in defining reactive arthritis (ReA) across the included studies. The absence of uniform diagnostic criteria for ReA introduces challenges in accurately assessing disease incidence and its association with specific bacterial infections. This variability in definition may have influenced the interpretation of our meta-analysis results and underscores the need for standardized diagnostic guidelines in future research.

All included studies were mainly from European and Western countries. Thus, the meta-analysis findings may not be directly applicable or fully transferable to populations in Asian and African countries. This systematic review and meta-analysis revealed the presence of publication bias that might negatively affect the reliability and validity of the findings. Finally, the study considered only research publications written in the English language, which could introduce language bias into the findings.

The spectrum of manifestations observed in reactive arthritis (ReA), as reported in the original papers analyzed in our study, demonstrated significant clinical diversity. While certain studies delineated the classic triad of symptoms—arthritis, urethritis, and conjunctivitis—following bacterial infections, others presented a wide array of clinical scenarios with varying degrees of joint involvement and extra-articular symptoms. Additionally, some studies reported the presence of joint pain without specifying the location or duration of the disease at the time of assessment. This observed variability underscores the inherently heterogeneous nature of ReA, posing challenges for its diagnosis and management. Such diversity necessitates a nuanced approach to understanding and addressing the complexities of ReA diagnosis, treatment, and patient care.

Given the heterogeneity in the systematic review and meta-analysis, a sensitivity analysis is necessary to address this issue. One can determine how much these outlier studies affect the overall effect size by excluding outliers or conducting analyses with and without outlier studies. However, further research is needed to determine the proportions of *Campylobacteria*, *Escherichia*, *Salmonella*, *Shigella*, and *Yersinia* strains responsible for causing reactive arthritis in Asian and African countries. Such research would improve our understanding of the disease’s prevalence in these regions.

In summary, our meta-analysis revealed significant variability in the included reports of discrete bacterial infections associated with ReA. This diversity underscores challenges in ReA epidemiology due to underreporting, regional variations, and differing levels of research focused on specific pathogens. Future studies should prioritize targeted surveillance to better elucidate the true burden of ReA across different geographic contexts and populations.

## Data Availability

All the data generated or analyzed during this study are included in this published article (and its Supplementary Materials).

## Supplementary Materials

The following supporting information can be downloaded https://www.mdpi.com/article/10.3390/jcm13123433/s1, Figure S1: Funnel plot for all the studies included; Figure S2: Cumulative meta-analysis of *Campylobacter* studies; Figure S3: Cumulative meta-analysis of *Escherichia* studies; Figure S4: Cumulative meta-analysis of *Salmonella* studies; Figure S5: Cumulative meta-analysis of *Shigella* studies; Figure S6: Cumulative meta-analysis of *Yersinia* studies; Table S1: Keyword combinations used for literature search in databases; Table S2: PRISMA checklist; Table S3: Primary literature search screening results for *Campylobacter* studies; Table S4: Primary literature search screening results for *Escherichia* studies; Table S5: Primary literature search screening results for *Salmonella* studies; Table S6: Primary literature search screening results for *Shigella* studies; Table S7: Primary literature search screening results for *Yersinia* studies; Table S8: Trim and fill analysis results for *Campylobacter* infection; Table S9: Trim and fill analysis results for *Escherichia* infection; Table S10: Trim and fill analysis results for *Salmonella* infection; Table S11: Trim and fill analysis results for *Shigella* infection; Table S12: Trim and fill analysis.
