# Supplementary material for: Enteric Infection-Associated Reactive Arthritis: A Systematic Review and Meta-Analysis
Source: J Clin Med. 2024 Jun 12;13(12):3433. doi: 10.3390/jcm13123433 (PMC11205162; doi:10.3390/jcm13123433)
Supplement: Supplementary file 1 [file jcm-13-03433-s001.zip › jcm-3020022-supplementary.pdf]

Supplementary Materials

Table S1 – Keyword combinations used for literature search in databases

Bacteria	PubMed	Embase	Scopus	Web of Science
Campylobacter	(arthritis, reactive[MeSH Terms]) AND (campylobacter[MeSH Terms])	('reactive arthritis'/exp OR 'reactive arthritis') AND ('campylobacter'/exp OR campylobacter)	TITLE-ABS-KEY ({reactive arthritis} OR {reactive arthritides} OR {post-infectious arthritis} OR {post infectious arthritis} OR {postinfectious arthritis} OR {arthritis post-infectious} OR {arthritides post-infectious} OR {arthritis post infectious} OR {post-infectious arthritides} OR {arthritis postinfectious} OR {arthritides postinfectious} OR {postinfectious arthritides} OR {reiter syndrome} OR {syndrome reiter} OR {reiter's disease} OR {disease reiter's} OR {reiters disease} OR {reiter disease} OR {disease reiter} AND {campylobacter})	(ALL=("reactive arthritis") AND ALL=(campylobacter))
Escherichia	(arthritis, reactive[MeSH Terms]) AND (escherichia [MeSH Terms])	('reactive arthritis'/exp OR 'reactive arthritis') AND (' escherichia '/exp OR escherichia)	TITLE-ABS-KEY ({reactive arthritis} OR {reactive arthritides} OR {post-infectious arthritis} OR {post infectious arthritis} OR {postinfectious arthritis} OR {arthritis post-infectious} OR {arthritides post-infectious} OR {arthritis post infectious} OR {post-infectious arthritides} OR {arthritis postinfectious} OR {arthritides postinfectious} OR {postinfectious arthritides} OR {reiter syndrome} OR {syndrome reiter} OR {reiter's disease} OR {disease reiter's} OR {reiters disease} OR {reiter	(ALL=("reactive arthritis") AND ALL=(escherichia))

			disease} OR {disease reiter} AND {escherichia})	
Salmonella	(arthritis, reactive[MeSH Terms]) AND (salmonella [MeSH Terms	('reactive arthritis'/exp OR 'reactive arthritis') AND ('salmonella'/exp OR salmonella)	TITLE-ABS-KEY ({reactive arthritis} OR {reactive arthritides} OR {post-infectious arthritis} OR {post infectious arthritis} OR {postinfectious arthritis} OR {arthritis post-infectious} OR {arthritides post-infectious} OR {arthritis post infectious} OR {post-infectious arthritides} OR {arthritis postinfectious} OR {arthritides postinfectious} OR {postinfectious arthritides} OR {reiter syndrome} OR {syndrome reiter} OR {reiter's disease} OR {disease reiter's} OR {reiters disease} OR {reiter disease} OR {disease reiter} AND {salmonella})	(ALL=("reactive arthritis") AND ALL=(salmonella))
Shigella	(reactive arthritis[MeSH Terms]) AND (shigella[MeSH Terms])	('reactive arthritis'/exp OR 'reactive arthritis') AND ('shigella'/exp OR 'shigella')	TITLE-ABS-KEY ({reactive arthritis} OR {reactive arthritides} OR {post-infectious arthritis} OR {post infectious arthritis} OR {postinfectious arthritis} OR {arthritis post-infectious} OR {arthritides post-infectious} OR {arthritis post infectious} OR {post-infectious arthritides} OR {arthritis postinfectious} OR {arthritides postinfectious} OR {postinfectious arthritides} OR {reiter syndrome} OR {syndrome reiter} OR {reiter's disease} OR {disease reiter's} OR {reiters disease} OR {reiter disease} OR {disease reiter} AND {shigella} OR {shigella dysenteriae} OR {shigella flexneri} OR {shigella sonnei})	(ALL=("reactive arthritis") AND ALL=(shigella))

Yersinia	(reactive arthritis[MeSH Terms]) AND (yersinia[MeSH Terms])	('reactive arthritis'/exp OR 'reactive arthritis') AND ('yersinia'/exp OR yersinia)	TITLE-ABS-KEY ({reactive arthritis} OR {reactive arthritides} OR {post-infectious arthritis} OR {post infectious arthritis} OR {postinfectious arthritis} OR {arthritis post-infectious} OR {arthritides post-infectious} OR {arthritis post infectious} OR {post-infectious arthritides} OR {arthritis postinfectious} OR {arthritides postinfectious} OR {postinfectious arthritides} OR {reiter syndrome} OR {syndrome reiter} OR {reiter's disease} OR {disease reiter's} OR {reiters disease} OR {reiter disease} OR {disease reiter} AND {yersinia} OR {yersinia pseudotuberculosis} OR {yersinia enterocolitica})	(ALL=("reactive arthritis") AND ALL=(yersinia))

Table S2 – PRISMA checklist

Section and Topic	Item #	Checklist item	Location where item is reported
**TITLE**			
Title	1	Identify the report as a systematic review.	1
**ABSTRACT**			
Abstract	2	See the PRISMA 2020 for Abstracts checklist.	1
**INTRODUCTION**			
Rationale	3	Describe the rationale for the review in the context of existing knowledge.	1
Objectives	4	Provide an explicit statement of the objective(s) or question(s) the review addresses.	1
**METHODS**			
Eligibility criteria	5	Specify the inclusion and exclusion criteria for the review and how studies were grouped for the syntheses.	2
Information sources	6	Specify all databases, registers, websites, organisations, reference lists and other sources searched or consulted to identify studies. Specify the date when each source was last searched or consulted.	2
Search strategy	7	Present the full search strategies for all databases, registers and websites, including any filters and limits used.	Supplementary data, table S1
Selection process	8	Specify the methods used to decide whether a study met the inclusion criteria of the review, including how many reviewers screened each record and each report retrieved, whether they worked independently, and if applicable, details of automation tools used in the process.	2, 3
Data collection process	9	Specify the methods used to collect data from reports, including how many reviewers collected data from each report, whether they worked independently, any processes for obtaining or confirming data from study investigators, and if applicable, details of automation tools used in the process.	2, 3
Data items	10a	List and define all outcomes for which data were sought. Specify whether all results that were compatible with each outcome domain in each study were sought (e.g. for all measures, time points, analyses), and if not, the methods used to decide which results to collect.	6-8, 11-12, 17-21, 25-26, 30-31
	10b	List and define all other variables for which data were sought (e.g. participant and intervention characteristics, funding sources). Describe any assumptions made about any missing or unclear information.	4, 6-8, 9-12, 14,17-21, 23, 25-28, 30-31
Study risk of bias assessment	11	Specify the methods used to assess risk of bias in the included studies, including details of the tool(s) used, how many reviewers assessed each study and whether they worked independently, and if applicable, details of automation tools used in the process.	5, 10, 15, 23, 28
Effect measures	12	Specify for each outcome the effect measure(s) (e.g. risk ratio, mean difference) used in the synthesis or presentation of results.	9, 14, 22, 27, 32
Synthesis methods	13a	Describe the processes used to decide which studies were eligible for each synthesis (e.g. tabulating the study intervention characteristics and comparing against the planned groups for each synthesis (item #5)).	2, 3
	13b	Describe any methods required to prepare the data for presentation or synthesis, such as handling of missing summary statistics, or data	4

Section and Topic	Item #	Checklist item	Location where item is reported
		conversions.	
	13c	Describe any methods used to tabulate or visually display results of individual studies and syntheses.	4
	13d	Describe any methods used to synthesize results and provide a rationale for the choice(s). If meta-analysis was performed, describe the model(s), method(s) to identify the presence and extent of statistical heterogeneity, and software package(s) used.	4
	13e	Describe any methods used to explore possible causes of heterogeneity among study results (e.g. subgroup analysis, meta-regression).	4-5, 9-11, 14-16, 22-24, 27-29
	13f	Describe any sensitivity analyses conducted to assess robustness of the synthesized results.	33
Reporting bias assessment	14	Describe any methods used to assess risk of bias due to missing results in a synthesis (arising from reporting biases).	5, 10, 15, 23, 28
Certainty assessment	15	Describe any methods used to assess certainty (or confidence) in the body of evidence for an outcome.	
**RESULTS**			
Study selection	16a	Describe the results of the search and selection process, from the number of records identified in the search to the number of studies included in the review, ideally using a flow diagram.	2, 3
	16b	Cite studies that might appear to meet the inclusion criteria, but which were excluded, and explain why they were excluded.	Supplementary data, table S3-S7
Study characteristics	17	Cite each included study and present its characteristics.	6-8, 12-13, 17-21, 25-26, 30-31
Risk of bias in studies	18	Present assessments of risk of bias for each included study.	Supplementary data, table S3-S7
Results of individual studies	19	For all outcomes, present, for each study: (a) summary statistics for each group (where appropriate) and (b) an effect estimate and its precision (e.g. confidence/credible interval), ideally using structured tables or plots.	9, 14, 22, 27, 32
Results of syntheses	20a	For each synthesis, briefly summarise the characteristics and risk of bias among contributing studies.	4-5, 9, 10, 14, 15, 22, 23, 27, 28, 32
	20b	Present results of all statistical syntheses conducted. If meta-analysis was done, present for each the summary estimate and its precision (e.g. confidence/credible interval) and measures of statistical heterogeneity. If comparing groups, describe the direction of the effect.	4-5, 9, 10, 14, 15, 22, 23, 27, 28, 32
	20c	Present results of all investigations of possible causes of heterogeneity among study results.	4-5, 9-10, 14-15, 22-23, 27-28, 32-33

Section and Topic	Item #	Checklist item	Location where item is reported
	20d	Present results of all sensitivity analyses conducted to assess the robustness of the synthesized results.	33
Reporting biases	21	Present assessments of risk of bias due to missing results (arising from reporting biases) for each synthesis assessed.	Supplementary data, table S3-S7
Certainty of evidence	22	Present assessments of certainty (or confidence) in the body of evidence for each outcome assessed.	4-5, 9-10, 14-15, 22-23, 27-28
**DISCUSSION**			
Discussion	23a	Provide a general interpretation of the results in the context of other evidence.	32-33
	23b	Discuss any limitations of the evidence included in the review.	33
	23c	Discuss any limitations of the review processes used.	32-33
	23d	Discuss implications of the results for practice, policy, and future research.	33
**OTHER INFORMATION**			
Registration and protocol	24a	Provide registration information for the review, including register name and registration number, or state that the review was not registered.	N/A
	24b	Indicate where the review protocol can be accessed, or state that a protocol was not prepared.	2-3
	24c	Describe and explain any amendments to information provided at registration or in the protocol.	2-3
Support	25	Describe sources of financial or non-financial support for the review, and the role of the funders or sponsors in the review.	33
Competing interests	26	Declare any competing interests of review authors.	33
Availability of data, code and other materials	27	Report which of the following are publicly available and where they can be found: template data collection forms; data extracted from included studies; data used for all analyses; analytic code; any other materials used in the review.	N/A

Table S3 – Primary literature search screening results for *Campylobacter* studies

Campylobacter

№	Authors	Year	Comments	Results
1	Ang et al.	2007	Validation of diagnostic method	Excluded
2	Arai et al.	2007	Case report, wrong sequelae	Excluded
3	Bengtsson et al.	1983	Wrong study design	Excluded
4	Berden et al.	1979	Case report	Excluded
5	Bremell et al.	1991		Included
6	Chou et al.	1998	Antibodies detection in current RA, AS patients	Excluded
7	Coustilleres et al.	2022	Case report, wrong sequelae	Excluded
8	Cristea et al.	2019	Validation of diagnostic method	Excluded
9	Curry et al.	2010	Wrong study design	Excluded
10	Doorduyn et al.	2008		Included
11	Dubey et al.	2021	Disease mechanism investigation	Excluded
12	Eastmond et al.	1983		Included
13	Ebright & Ryan	1984	Case report	Excluded
14	Engberg et al.	2003	Validation of diagnostic method	Excluded
15	Erlacher et al.	1995	Validation of diagnostic method	Excluded
16	Esan et al.	2020	Wrong study design	Excluded
17	Fryden et al.	1990	Treatment assessment study	Excluded
18	Garg et al.	2008	Wrong sequelae	Excluded
19	Gibney et al.	2014	Wrong study design	Excluded
20	Gumpel et al.	1981		Included
21	Hannu et al.	2002		Included
22	Hannu et al.	2004		Included
23	Hassel et al.	1994	Disease mechanism investigation	Excluded
24	Helms et al.	2006		Included
25	Henry et al.	2000	Wrong sequelae	Excluded
26	Johnsen et al.	1983		Included
27	Kosunen et al.	1981		Included

28	Kumagai et al.	2015	Wrong study design, epidemiological study	Excluded
29	Lackner et al.	2019	Wrong sequelae	Excluded
30	Laine et al.	2014	Wrong sequelae	Excluded
31	Lapadula et al.	1992	Antibodies detection in current ReA patients	Excluded
32	Latha et al.	2017	Wrong sequelae	Excluded
33	Leung et al.	1980	Case report	Excluded
34	Locht & Krogfelt	2002		Included
35	Lorente et al.	2020	Disease mechanism investigation	Excluded
36	Lund et al.	2015	Wrong sequelae	Excluded
37	Majowicz et al.	2020	Wrong study design, epidemiological study	Excluded
38	Mäki-Ikola et al.	1991	Antibodies detection in current arthritis patients	Excluded
39	Melby et al.	2000		Included
40	Mellou & Velonakis	2011	Wrong study design, epidemiological study	Excluded
41	Mihalič & Trebše	2020	Case report	Excluded
42	Miljkovic-Selimovic et al.	2010	Bacterial strain characterization	Excluded
43	Mortensen et al.	2009	Disease mechanism investigation	Excluded
44	Nielsen et al.	2010	Validation of diagnostic method	Excluded
45	Nielsen et al.	2012	Disease mechanism investigation	Excluded
46	Ninell et al.	2009	Disease mechanism investigation	Excluded
47	Petersen et al.	1996		Included
48	Pitkänen et al.	1981		Included
49	Pitkänen et al.	1983		Included
50	Pönkä et al.	1984		Included
51	Porter et al.	2013		Included
52	Rees et al.	2004		Included
53	Ruzante et al.	2011	Wrong study design, epidemiological study	Excluded
54	Scallan et al.	2015	Wrong study design, epidemiological study	Excluded
55	Schiellerup et al.	2008		Included

56	Schmidt-Ott et al.	2005	Validation of diagnostic method	Excluded
57	Schönberg-Norio et al.	2010		Included
58	Schorling et al.	2023	Wrong study design, epidemiological study	Excluded
59	Short et al.	1982	Case report	Excluded
60	Smith	2002	Wrong sequelae	Excluded
61	Söderlin et al.	2003	Current ReA patients tested for bacteria	Excluded
62	Ternhag et al.	2008		Included
63	Townes et al.	2008		Included
64	van de Putte et al.	1980	Wrong study design, small cohort	Excluded
65	Verma et al.	2020	Disease mechanism investigation	Excluded
66	Walker et al.	2022		Included
67	Weir et al.	1979	Case report	Excluded
68	Zautner et al.	2014	Validation of diagnostic method	Excluded
69	Zia et al.	2003		Included

Table S4 – Primary literature search screening results for *Escherichia* studies

Escherichia

№	Authors	Year	Comments	Results
1	Adam et al.	2003	Disease mechanism investigation	Excluded
2	Chen et al.	2002	RA disease mechanism investigation	Excluded
3	Chou et al.	1998	Antibodies detection in current AS, RA patients	Excluded
4	Garg et al.	2008	Wrong sequelae	Excluded
5	Helms et al.	2006		Included
6	Houtman et al.	2012	Validation of diagnostic method	Excluded
7	Laasila et al.	1999	Wrong sequelae	Excluded
8	Lee et al.	2023	Wrong sequelae	Excluded
9	Locht & Krogfelt	2002		Included
10	Madhavan et al.	2002	Antibodies detection in current AS patients	Excluded

11	Makiikola et al.	1994	Antibodies detection in current AS patients	Excluded
12	Nishizaki et al.	2016	Case report, wrong sequelae	Excluded
13	Rees et al.	2004		Included
14	Sangha et al.	2018	Wrong study design	Excluded
15	Schiellerup et al.	2008		Included
16	Siala et al.	2009	Validation of diagnostic method	Excluded
17	Renou et al.	2011	Case report	Excluded
18	Ruzante et al.	2011	Wrong study design, epidemiological study	Excluded
19	Ternhag et al.	2008		Included
20	Thomas et al.	2003	Wrong study design, epidemiological study	Excluded
21	Townes et al.	2008		Included
22	Tuompo et al.	2020		Included
23	Zhao et al.	2023	Case report, wrong sequelae	Excluded

Table S5 – Primary literature search screening results for *Salmonella* studies

Salmonella

№	Authors	Year	Comments	Results
1	Antoniou et al.	2019	Disease mechanism investigation	Excluded
2	Arnedo-Pena et al.	2010		Included
3	Ananthanathorn et al.	2022	Wrong sequelae	Excluded
4	Bengtsson et al.	1955		Included
5	Briem et al.	1978	Case report	Excluded
6	Brodie & Ehresmann	1983	Wrong sequelae, case report	Excluded
7	Buxton et al.	2002		Included
8	Carroll et al.	1981	Case report	Excluded
9	Chaurasia et al.	2016	Disease mechanism investigation	Excluded
10	Constantiniu et al.	2008	Antibodies detection in current ReA patients	Excluded
11	Doorduyn et al.	2008		Included
12	Dworkin et al.	2001		Included
13	Eastmond et al.	1983		Included

14	Ekman et al.	2000		Included
15	Esan et al.	2020	Wrong study design	Excluded
16	Ford et al.	2019	Wrong study design, epidemiological study	Excluded
17	Gibney et al.	2014	Wrong study design	Excluded
18	Golding & Robertson	1985	Case report	Excluded
19	Håkansson et al.	1976		Included
20	Hannu et al.	2002		Included
21	Helms et al.	2006		Included
22	Herrero-Beaumont et al.	1990	Case reports	Excluded
23	Hermann et al.	1993	Disease mechanism investigation	Excluded
24	Huppertz et al.	1995	Case reports	Excluded
25	Inman et al.	1988		Included
26	Iveson et al.	1975	Clinical cases description	Excluded
27	Jones et al.	1977	Case report	Excluded
28	Kanakoudi-Tsakalidou et al.	1998	Clinical cases description	Excluded
29	Kondowe et al.	1989	Wrong study design	Excluded
30	Lacoste et al.	2007	Case report	Excluded
31	Lahesmaa et al.	1991	Disease mechanism investigation	Excluded
32	Lee et al.	2005		Included
33	Locht et al.	1993		Included
34	Mäki-Ikola et al.	1991	Antibodies detection in current ReA patients	Excluded
35	Mattila et al.	1994		Included
36	Mattila et al.	1998		Included
37	McColl et al.	2000		Included
38	Meyer-Bahlburg et al.	2001	Disease mechanism investigation	Excluded
39	Nikkari et al.	1999	Validation of diagnostic method	Excluded
40	Ortiz-Alvarez et al.	1998	Disease mechanism investigation	Excluded
41	Petersen et al.	1996		Included
42	Porter et al.	2013		Included
43	Puddey et al.	1982	Case report	Excluded
44	Rees et al.	2004		Included
45	Rohekar et al.	2008		Included
46	Rudwaleit et al.	2001		Included
47	Ruzante et al.	2011	Wrong study design, epidemiological study	Excluded

48	Samuel et al.	1995		Included
49	Saxena et al.	2005	Disease mechanism investigation	Excluded
50	Schiellerup et al.	2008		Included
51				
52	Sieper et al.	1999	Clinical trials	Excluded
53	Singh et al.	2007	Disease mechanism investigation	Excluded
54	Singh et al.	2013	Disease mechanism investigation	Excluded
55	Sinha et al.	2003	Disease mechanism investigation	Excluded
56	Sobieszczańska & Przondo-Mordarska	1996	Disease mechanism investigation, antibodies detection	Excluded
57	Söderlin et al.	2003	Current ReA patients testing for bacteria	Excluded
58	Ringrose et al.	2004	Disease mechanism investigation	Excluded
59	Ternhag et al.	2008		Included
60	Thomas & Hedayati	1986	Case report	Excluded
61	Thomson et al.	1992		Included
62	Thomson et al.	1994		Included
63	Thomson et al.	1995		Included
64	Townes et al.	2008		Included
65	Trull et al.	1986	Antibodies detection in current ReA patients	Excluded
66	Tuompo et al.	2013		Included
67	Urfer et al.	2000		Included
68	Vuento et al.	1983	Disease mechanism investigation	Excluded
69	Warren	1970	Case reports	Excluded
70	Weiss et al.	1980	Case report	Excluded
71	Wilson & Whitehead	2004	Case report	Excluded
72	Zhao et al.	2017	Disease mechanism investigation	Excluded

Table S6 – Primary literature search screening results for *Shigella* studies

Shigella

№	Authors	Year	Comments	Results

1	Brandt et al.	2004	Disease mechanism investigation	Excluded
2	Chen et al.	2002	Case report	Excluded
3	Constantiniu et al.	2008	Antibodies detection in current ReA patients	Excluded
4	Cristea et al.	2015	Disease mechanism investigation	Excluded
5	Fendler et al.	2001	Current ReA patients testing for bacteria	Excluded
6	Finch et al.	1986		Included
7	Good & Schultz	1977	Wrong study design	Excluded
8	Hannu et al.	2005		Included
9	Helms et al.	2006		Included
10	Inman & Chiu	2003	Disease mechanism investigation	Excluded
11	Islam et al.	1997	Wrong sequelae	Excluded
12	Kaslow et al.	1979		Included
13	Kennedy et al.	2017	Case report	Excluded
14	Lauhio et al.	1988	Case reports	Excluded
15	Lewis	1982	Letter to editor	Excluded
16	Martin et al.	2012		Included
17	Mazumder et al.	1997	Case report	Excluded
18	McGuire et al.	2019	Wrong study design, epidemiological study	Excluded
19	Morse et al.	1980	Wrong study design	Excluded
20	Noer	1966		Included
21	Petersen et al.	1996		Included
22	Porter et al.	2013		Included
23	Raybourne et al.	1988	Disease mechanism investigation	Excluded
24	Rees et al.	2004		Included
25	Salas-Cuestas et al.	2017	Wrong study design, antibodies detection in current ReA patients	Excluded
26	Schiellerup et al.	2008		Included
27	Siala et al.	2008	Bacterial DNA detection in synovial tissue of current ReA patients	Excluded
28	Siala et al.	2018		Excluded
29	Sieper et al.	1993	Disease mechanism investigation	Excluded
30	Simon et al.	1981		Included

31	Sobieszczańska & Przondo-Mordarska	1996	Disease mechanism investigation, antibodies detection	Excluded
32	Stieglitz & Lipsky	1993	Disease mechanism investigation	Excluded
33	Ternhag et al.	2008		Included
34	Townes et al.	2008		Included
35	Tsuchiya et al.	1990	Disease mechanism investigation, antibodies detection	Excluded
36	van Bohemen et al.	1986		Included
37	Zwillich et al.	1989	Disease mechanism investigation	Excluded

Table S7 – Primary literature search screening results for *Yersinia* studies

Yersinia

№	Authors	Year	Comments	Results
1	Bengtsson et al.	1983	Wrong study design	Excluded
2	Borg et al.	1992	Literature review	Excluded
3	Constantiniu et al.	1989	Antibodies detection in current ReA patients	Excluded
4	Constantiniu et al.	2008	Antibodies detection in current ReA patients	Excluded
5	Fendler et al.	2001	Current ReA patients testing for bacteria	Excluded
6	Gaston et al.	2001	Case report	Excluded
7	Granfors et al.	1998	Disease mechanism investigation	Excluded
8	Granfors et al.	2008	Wrong study design	Excluded
9	Hannu et al.	2003		Included
10	Helms et al.	2006		Included
11	Herrlinger et al.	1992	Antibodies detection in current ReA patients	Excluded
12	Honda et al.	2017	Case report	Excluded
13	Huovinen et al.	2010		Included
14	Kobayashi et al.	1985	Antibodies detection in current ReA patients	Excluded
15	Kono et al.	1984	Antibodies detection in current ReA, AS patients	Excluded
16	Lacoste et al.	2007	Wrong study design	Excluded

17	Leirisalo-Repo et al.	1987	Wrong study design	Excluded
18	Lindley et al.	1989	Case report	Excluded
19	Locht et al.	1995	Antibodies detection in current ReA patients	Excluded
20	Long et al.	2010	Letter to the editor	Excluded
21	Mäki-Ikola et al.	1994	Antibodies detection in current ReA patients	Excluded
22	Mertz et al.	2004	Disease mechanism investigation	Excluded
23	Nikkari et al.	1992	Validation of diagnostic method	Excluded
24	Petersen et al.	1996		Included
25	Porter et al.	2013		Included
26	Rees et al.	2004		Included
27	Rosner et al.	2013		Included
28	Saario et al.	1992	Wrong sequelae	Excluded
29	Schiellerup et al.	2008		Included
30	Schlaak et al.	1992	Disease mechanism investigation	Excluded
31	Sobieszczańska & Przondo-Mordarska	1996	Disease mechanism investigation, antibodies detection	Excluded
32	Söderlin et al.	2003	Current ReA patients testing for bacteria	Excluded
33	Ström & Johansson	1997	Case report	Excluded
34	Taccetti et al.	1994	Antibodies detection in current pediatric ReA patients	Excluded
35	Ternhag et al.	2008		Included
36	Tertti et al.	1984		Included
37	Tertti et al.	1989		Included
38	Townes et al.	2008		Included
39	Tuompo et al.	2017	Wrong study design	Excluded
40	Vasala et al.	2014		Included
41	Wakefield et al.	1989	Antibodies detection in current pediatric ReA, AS patients	Excluded

Table S8 – Trim and fill analysis results for *Campylobacter* infection

Studies	Logit proportion	95% conf. interval	
Observed	-3.546	-4.272	-2.820
Observed + Imputed	-3.987	-4.721	-3.252

Number of studies = 27
observed = 23
imputed = 4
Model: Random-effects
Method: REML

Figure S1 – Funnel plot for all the studies included

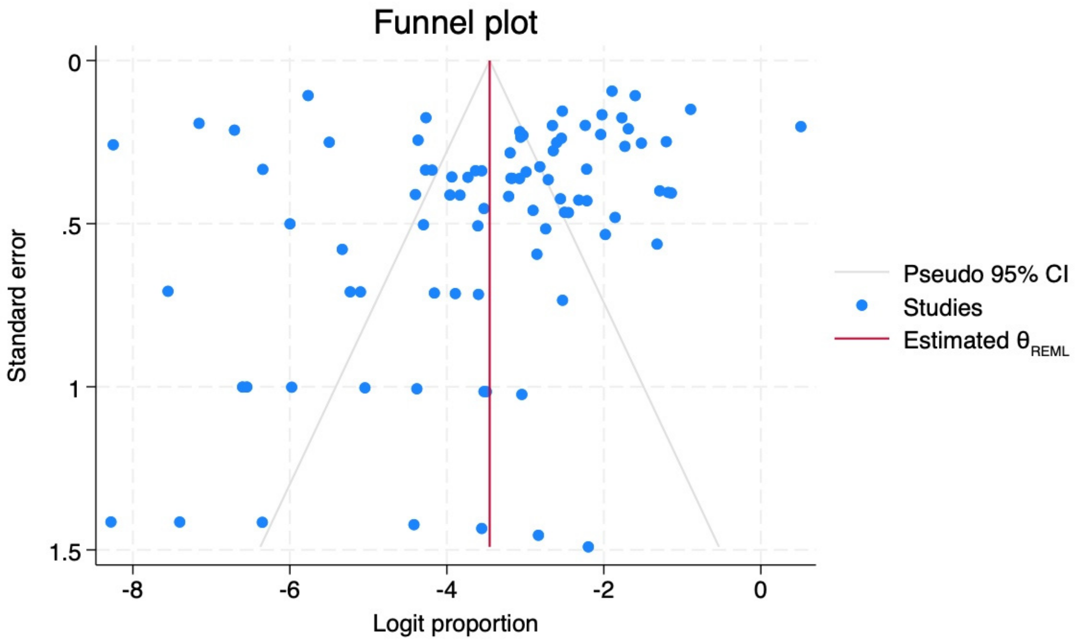

Figure S2 – Cumulative meta-analysis of *Campylobacter* studies

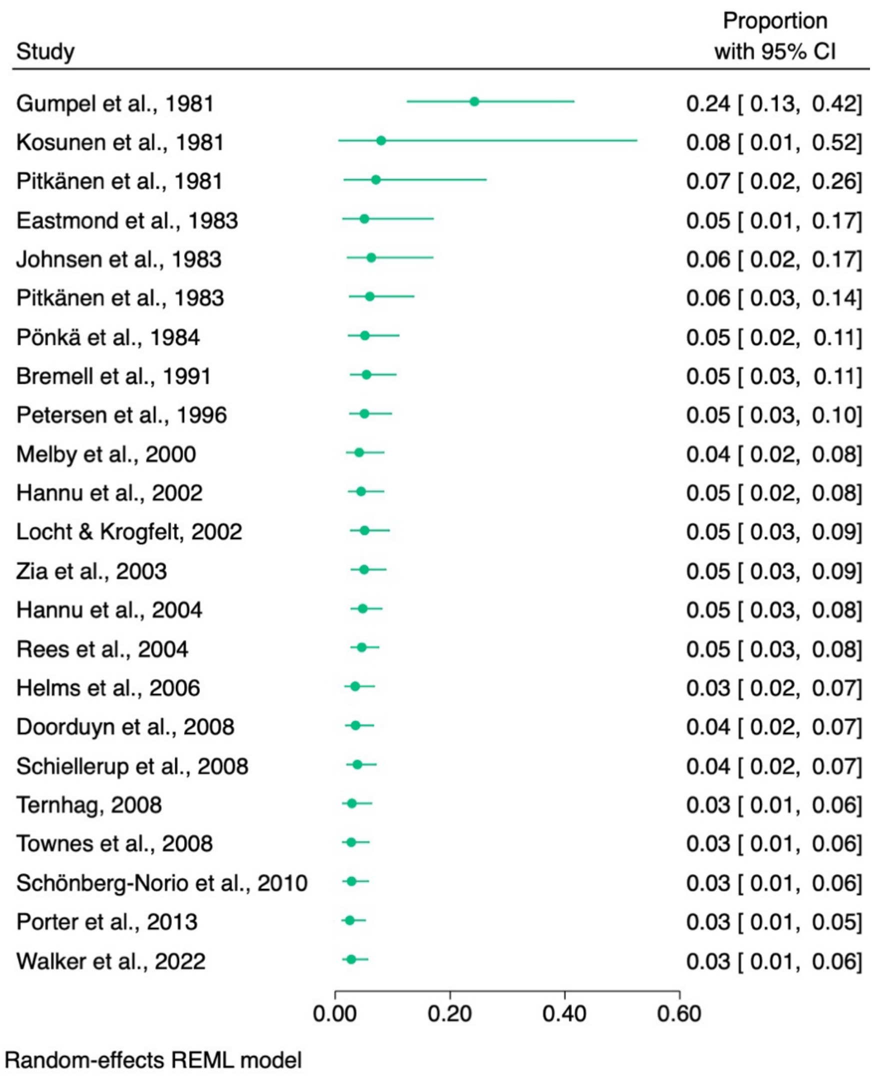

Table S9 – Trim and fill analysis results for *Escherichia* infection

Studies	Logit proportion	95% conf. interval	
Observed	-4.269	-5.723	-2.815
Observed + Imputed	-3.907	-5.416	-2.398

Number of studies = 8
observed = 7
imputed = 1
Model: Random-effects
Method: REML

Figure S3 – Cumulative meta-analysis of *Escherichia* studies

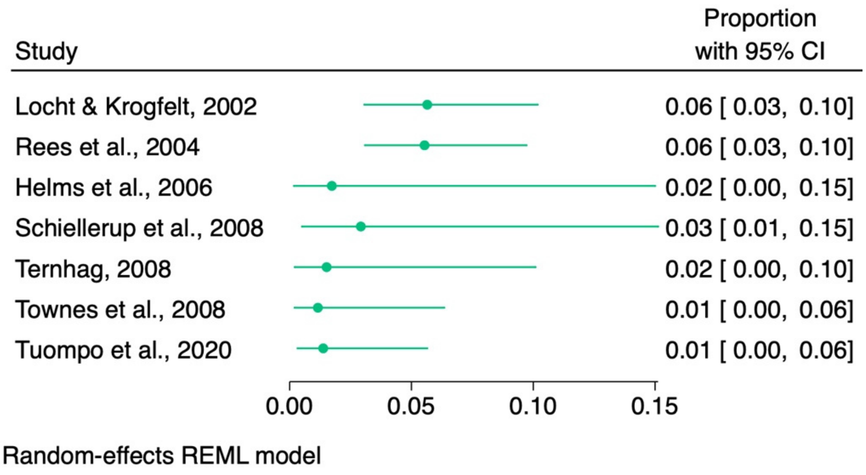

Table S10 – Trim and fill analysis results for *Salmonella* infection

Studies	Logit proportion	95% conf. interval	
Observed	-3.090	-3.669	-2.512
Observed + Imputed	-3.090	-3.669	-2.512

Number of studies = 30
observed = 30
imputed = 0
Model: Random-effects
Method: REML

Figure S4 – Cumulative meta-analysis of *Salmonella* studies

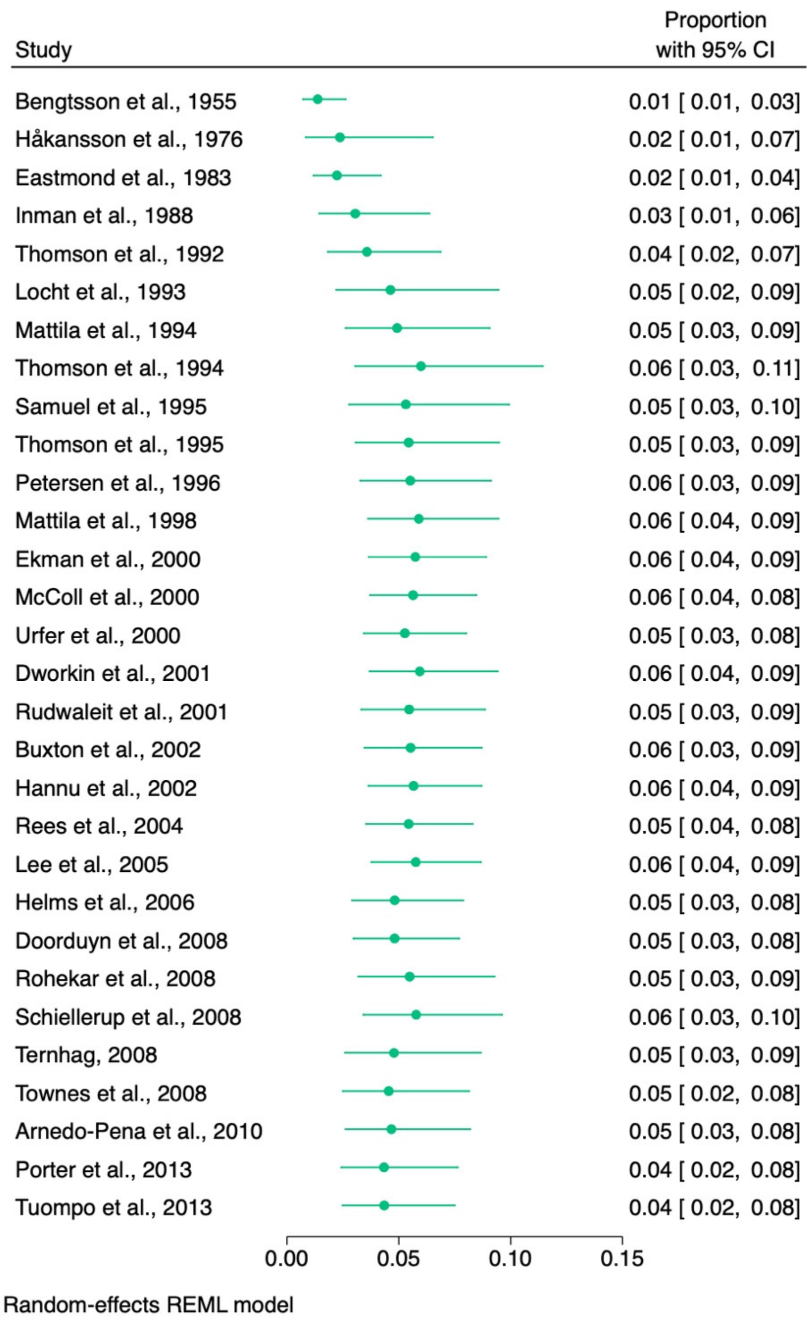

Table S11 – Trim and fill analysis results for *Shigella* infection

Studies	Logit proportion	95% conf. interval	
Observed	-4.292	-5.162	-3.422
Observed + Imputed	-4.292	-5.162	-3.422

Number of studies = 14
observed = 14

imputed = 0
Model: Random-effects
Method: REML

Figure S5 – Cumulative meta-analysis of *Shigella* studies

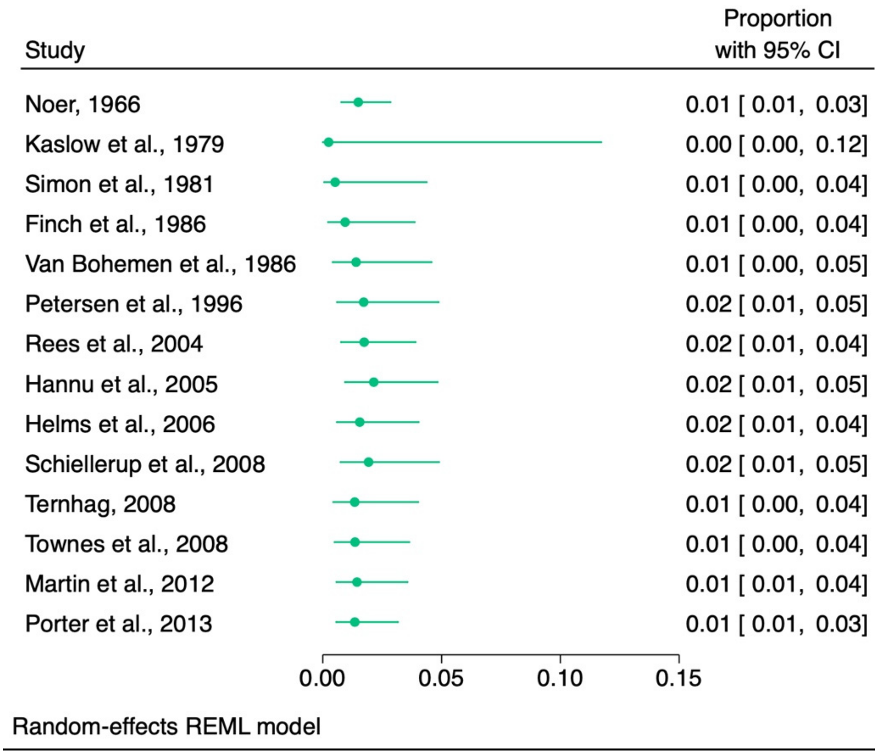

Table S12 – Trim and fill analysis results for *Yersinia* infection

Studies	Logit proportion	95% conf. interval	
Observed	-2.884	-3.849	-1.918
Observed + Imputed	-3.230	-4.161	-2.299

Number of studies = 15
observed = 13
imputed = 2
Model: Random-effects
Method: REML

Figure S6 – Cumulative meta-analysis of *Yersinia* studies

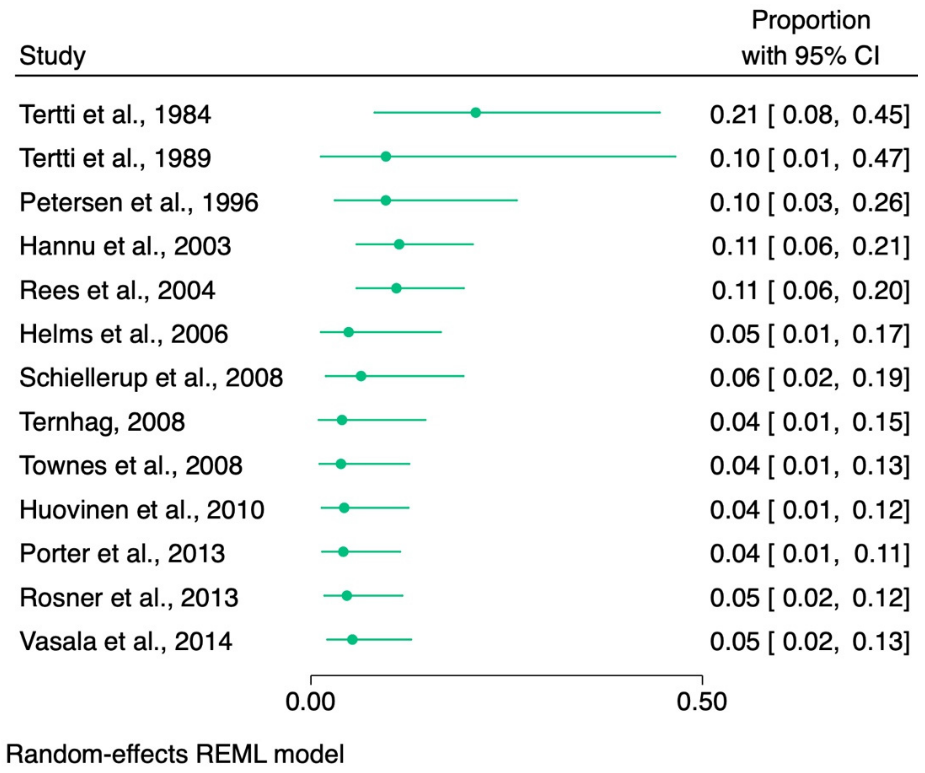
Study
Proportion with 95% CI
Tertti et al., 1984
0.21 [0.08, 0.45]
Tertti et al., 1989
0.10 [0.01, 0.47]
Petersen et al., 1996
0.10 [0.03, 0.26]
Hannu et al., 2003
0.11 [0.06, 0.21]
Rees et al., 2004
0.11 [0.06, 0.20]
Helms et al., 2006
0.05 [0.01, 0.17]
Schiellerup et al., 2008
0.06 [0.02, 0.19]
Ternhag, 2008
0.04 [0.01, 0.15]
Townes et al., 2008
0.04 [0.01, 0.13]
Huovinen et al., 2010
0.04 [0.01, 0.12]
Porter et al., 2013
0.04 [0.01, 0.11]
Rosner et al., 2013
0.05 [0.02, 0.12]
Vasala et al., 2014
0.05 [0.02, 0.13]
0.00
0.50
Random-effects REML model
